# Herpes Simplex Virus Type 1 Infection Disturbs the Mitochondrial Network, Leading to Type I Interferon Production through the RNA Polymerase III/RIG-I Pathway

**DOI:** 10.1128/mBio.02557-21

**Published:** 2021-11-23

**Authors:** Noémie Berry, Rodolphe Suspène, Vincent Caval, Pierre Khalfi, Guillaume Beauclair, Stéphane Rigaud, Hervé Blanc, Marco Vignuzzi, Simon Wain-Hobson, Jean-Pierre Vartanian

**Affiliations:** a Molecular Retrovirology Unit, Institut Pasteur, Paris, France; b Sorbonne Université, Complexité du Vivant, Paris, France; c Viral Genomics and Vaccination, Institut Pasteur, Paris, France; d Hub d’Analyse d’Images, Institut Pasteur, Paris, France; e Viral Populations and Pathogenesis Unit, Institut Pasteur, CNRS UMR 3569, Paris, France; Max Planck Unit for the Science of Pathogens

**Keywords:** HSV-1, mitochondria, innate immunity, cytidine deaminase, APOBEC3A, herpes simplex virus

## Abstract

Viruses have evolved a plethora of mechanisms to impair host innate immune responses. Herpes simplex virus type 1 (HSV-1), a double-stranded linear DNA virus, impairs the mitochondrial network and dynamics predominantly through the *UL12.5* gene. We demonstrated that HSV-1 infection induced a remodeling of mitochondrial shape, resulting in a fragmentation of the mitochondria associated with a decrease in their volume and an increase in their sphericity. This damage leads to the release of mitochondrial DNA (mtDNA) to the cytosol. By generating a stable THP-1 cell line expressing the DNase I-mCherry fusion protein and a THP-1 cell line specifically depleted of mtDNA upon ethidium bromide treatment, we showed that cytosolic mtDNA contributes to type I interferon and APOBEC3A upregulation. This was confirmed by using an HSV-1 strain (KOS37 UL98-SPA) with a deletion of the *UL12.5* gene that impaired its ability to induce mtDNA stress. Furthermore, by using an inhibitor of RNA polymerase III, we demonstrated that upon HSV-1 infection, cytosolic mtDNA enhanced type I interferon induction through the RNA polymerase III/RIG-I pathway. APOBEC3A was in turn induced by interferon. Deep sequencing analyses of cytosolic mtDNA mutations revealed an APOBEC3A signature predominantly in the 5′TpCpG context. These data demonstrate that upon HSV-1 infection, the mitochondrial network is disrupted, leading to the release of mtDNA and ultimately to its catabolism through APOBEC3-induced mutations.

## INTRODUCTION

Mitochondria participate in a broad range of innate immune pathways, functioning as a signaling platform and contributing to effector responses. Not surprisingly, they are the target of many different viral strategies to circumvent them (1). For example, Epstein-Barr virus encodes latent membrane protein 2A (LMP2A), leading to an increase of mitochondrial fission (2), and hepatitis C virus induces oxidative stress that damages mitochondria (3), while the hepatitis B virus X protein (HBx) induces apoptosis by causing a perinuclear clustering of mitochondria as well as the loss of the mitochondrial membrane potential (4). Recently, it was demonstrated that human influenza A virus encodes PB1-F2, which targets mitochondria, altering their morphology (5).

Herpes simplex virus type 1 (HSV-1) does not escape the rule. Indeed, HSV-1 infection is known to disorganize mitochondrial dynamics and significantly reduce ATP production within 12 h postinfection (6). This perturbation is predominantly executed by *UL12* (7, 8), which encodes two distinct yet similar proteins, UL12 and UL12.5. UL12 plays a crucial role in viral genome replication and processing, while the N-terminally truncated form, UL12.5, has nuclease and strand exchange activities and localizes predominantly to the mitochondria (9), where it triggers massive catabolism of mitochondrial DNA (mtDNA) during early HSV-1 replication (8). HSV-1 infection of mice results in the release of mtDNA to the cytosol and IFN induction through the cGAS/STING/IRF3 pathway (10), albeit without elimination of induction by other pathways. Infection of mice with an HSV-1 *UL12* mutant strain, deficient in mitochondrial targeting, failed to stimulate innate immunity, emphasizing the role of mtDNA in signaling (10).

The fact that cytosolic double-stranded DNA (dsDNA) is read as a danger signal is reflected in the large number of sensor molecules, such as RNA polymerase III, which transcribes DNA, leading to dsRNA intermediates that are subsequently captured by RIG-I, ultimately leading to type I interferon (IFN) production (11–13). Cytoplasmic DNases such as TREX1 and TREX2 degrade dsDNA into small single-stranded DNA (ssDNA) molecules of around 60 nucleotides (14). In placental mammals, these can be further degraded by APOBEC3 cytidine deamination, deuracilation by uracil *N*-glycosylase (UNG), and ssDNA cleavage by apyrimidine endonucleases (APE1 and APE2), which together result in catabolism of ssDNA. For humans, chromosome 22 harbors a cluster of 7 *APOBEC3* genes (*APOBEC3A*, *A3B*, *A3C*, *A3DE*, *A3F*, *A3G*, and *A3H*), encoding cytidine deaminases (15). All A3 enzymes, except A3DE, catalyze the deamination of cytidine to uracil on single-stranded DNA, leading to GC-to-AT editing (16–19). A3A, A3C, and A3H possess a nucleocytoplasmic localization, whereas A3DE, A3F, and A3G are found exclusively in the cytoplasm. As an exception, A3B is strictly nuclear. The remaining A3DE protein is catalytically inactive but can modulate A3F and A3G by heterodimer formation (20). Many of the corresponding genes are interferon-stimulated genes (ISGs), most notably *A3A*, which can be upregulated 10^4^-fold in primary leukocytes (13, 21–24).

A3A is so active that it can generate double-stranded-DNA breaks (DSBs) in an experimental setting and even edit mRNA at low levels (25–28). A3B can also edit nuclear DNA, albeit much less efficiently (25, 29). The role of A3A and A3B in cancer is highlighted by the fact that many cancer genomes encode tens of thousands of C-to-T transitions frequently in the 5′TpC dinucleotide context, a hallmark of A3A and A3B cytidine deamination (30–32). However, recent studies using high-throughput assays suggested that the preferred nucleotide motif for A3A is 5′TpCpG (33, 34).

We demonstrated here that HSV-1 infection in human acute monocytic leukemia cells triggers mitochondrial fragmentation and shapes the mitochondrial network by significantly reducing mitochondria volume. This leads to the release of mtDNA in the cytosol, inducing type I IFN through the RNA polymerase III/RIG-I pathway. A3A is in turn induced by IFN in a paracrine and autocrine manner to edit and shut down cytosolic mtDNA.

## RESULTS

### HSV-1 infection upregulates type I interferon and leads to *APOBEC3A* expression.

**FIG 1:**
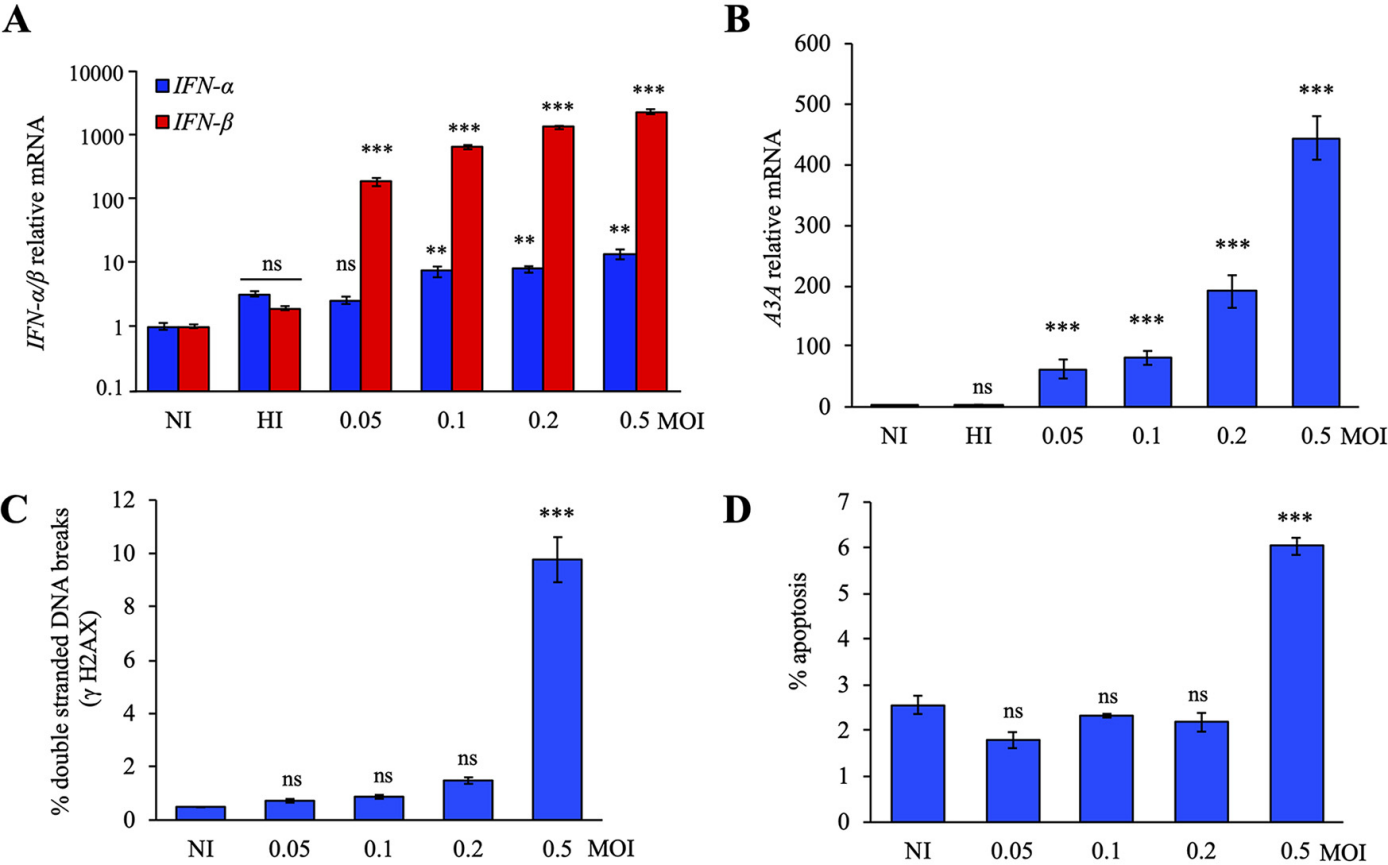
HSV-1 induced *APOBEC3A* upregulation. (A) Relative expression of *IFN-α* and *IFN-β* following HSV-1 infection at 18 h postinfection. Gene expression was normalized using the expression level of the housekeeping gene *RPL13A*. (B) *A3A* relative expression upon HSV-1 SC16 infection in THP-1 cells for 18 h postinfection. Mean values and standard deviations of the mean (SEM) were calculated for three independent infections in duplicate (*n* = 6). (C) Flow cytometry analysis of γH2AX positive cells at 18 h postinfection by HSV-1. (D) Flow cytometry analysis of annexin V-positive cells at 18 h postinfection by HSV-1. Mean values and SEM were calculated for three independent infections (*n* = 3). **, *P* < 0.05; ***, *P* < 0.005; ns, not significant (unpaired two-sided Student's *t* test).

To correlate the impact of viral stress on the induction of ISGs, we used the HSV-1 strain SC16 to infect THP-1 cells. This cell line was used because it is an IFN-inducible human acute monocytic leukemia cell line (13). At 18 h postinfection, infected cells were lysed, and total RNA was extracted and quantitated by TaqMan reverse transcription-PCR (RT-PCR). Different multiplicities of infection (MOI) of HSV-1 generated a strong *IFN-β* response in a dose-dependent manner, ∼160- to 2,000-fold greater than that in noninfected (NI) cells or THP-1 cells infected with heat-inactivated (HI) virus, but failed to induce potent induction of *IFN-α* expression (Fig. 1A).

Since some *A3* genes can be induced by type I IFN, a transcription study of *A3* genes was performed and showed that HSV-1 MOI increase was correlated with an increase in *A3A* expression at 18 h, in a dose-dependent manner, of ∼60- to 440-fold (Fig. 1B). Compared to the other *A3* genes, *A3A* upregulation was by far the most sensitive to HSV-1 infection, being upregulated by almost 2 orders of magnitude (Fig. S1A).

10.1128/mBio.02557-21.1 FIG S1 HSV-1 induced *APOBEC3A* and *IFN-α/β* upregulation. (A) *A3* upregulation upon HSV-1 infection in THP-1 cells for 18 h postinfection. Gene expression was normalized to the expression level of the housekeeping gene *RPL13A*. Differences between infected and noninfected cells were estimated with an unpaired two-sided Student’s t-test (***, *P* < 0.005). (B) *IFN* expression profiling upon HSV-1 infection of primary monocytes at 18 h postinfection, normalized to the expression levels of *RPL13A* and to the *IFN* expression obtained for noninfected cells. (C) *A3A* expression profiling upon HSV-1 infection of primary monocytes at 18 h postinfection, normalized to the expression levels of *RPL13A* and to *A3A* expression obtained for NI cells. Mean values and SEM were obtained from three independent experiments in duplicate (*n* = 6) (unpaired two-sided Student’s *t* test: **, *P* < 0.05; ***, *P* < 0.005). (D) *IFN-β* profiling by RT-qPCR at 24 h after transfection of 250 ng of DNA, at 18 h after infection with a heat-inactivated (HI) virus used as a negative control, or upon an infection with 0.2 MOI of HSV-1 in THP-1 cells expressing DNase I or not. Data were normalized to the *RPL13A* housekeeping gene. Mean values and SEM were calculated for three independent experiments in duplicate (*n* = 6) (unpaired two-sided Student’s *t* test: ***, *P* < 0.005; ns, not statistically significant). Download FIG S1, TIF file, 0.2 MB. Copyright © 2021 Berry et al. 2021 Berry et al. https://creativecommons.org/licenses/by/4.0/ This content is distributed under the terms of the Creative Commons Attribution 4.0 International license.

To further optimize our conditions of infection, THP-1 cells were infected for 18 h by HSV-1 at MOI from 0.05 to 0.5. As can be seen in Fig. 1C, only an MOI of 0.5 of HSV-1 generated DSBs in THP-1 cells ∼10-fold over background (NI cells), which was correlated with induction of apoptosis as assessed using annexin V staining (Fig. 1D). Thus, infections were performed over 18 h without exceeding an MOI of 0.5. To validate the effect of HSV-1 under more physiological conditions, primary monocytes isolated from healthy patient peripheral blood mononuclear cells (PBMCs) were infected with HSV-1 strain SC16 with a wider range of MOI, from 0.05 to 3. At 18 h postinfection, we observed a common overexpression of both *IFN-α* and *IFN-β* (Fig. S1B), leading to a dose-dependent upregulation of *A3A* expression (Fig. S1C).

### Cytosolic DNA is the source of IFN-β production leading to *APOBEC3A* upregulation.

**FIG 2:**
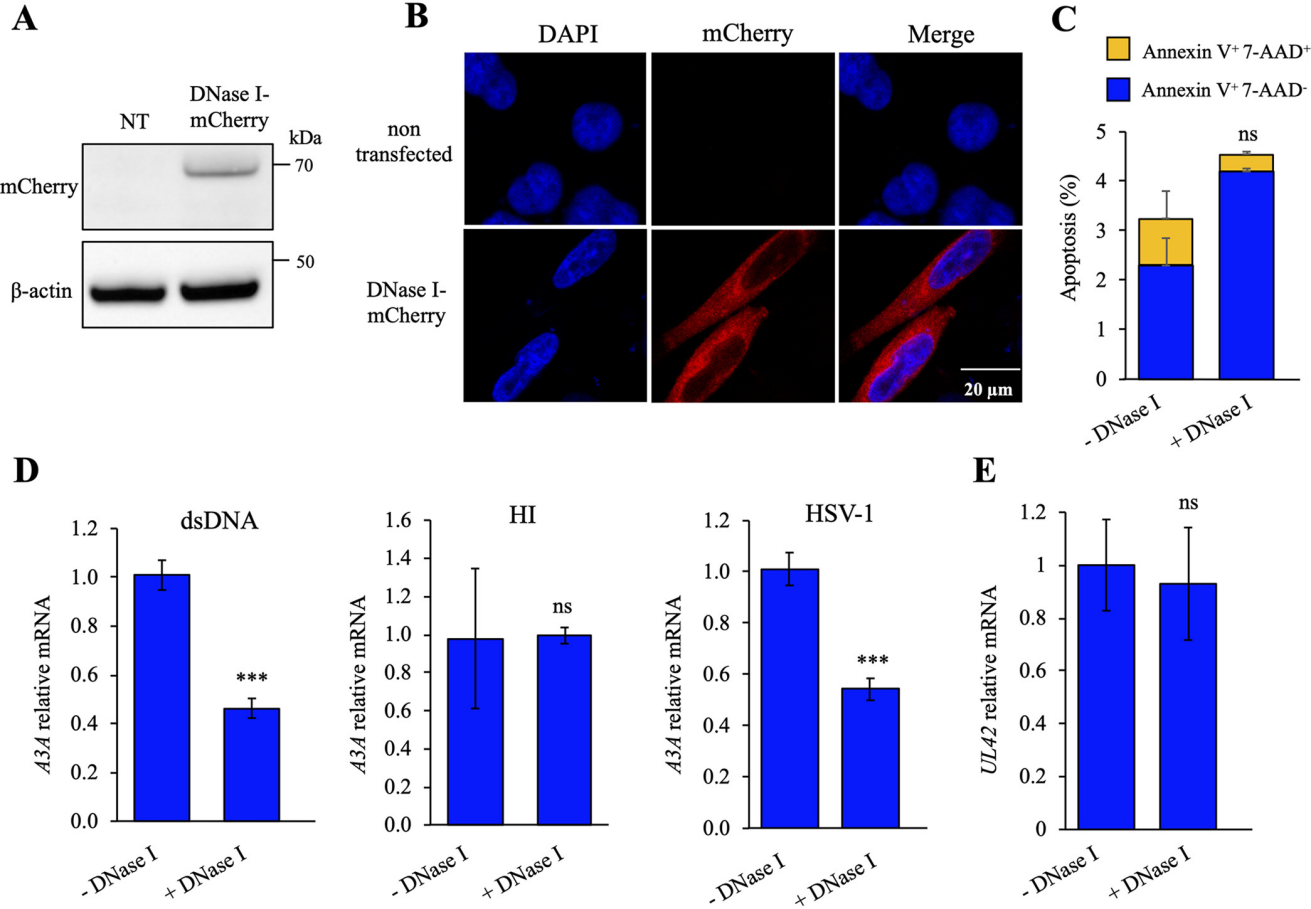
DNase I overexpression reduced *A3A* upregulation upon HSV-1 infection. Stable DNase I-overexpressing THP-1 cell lines were generated. (A) Western blot analysis of the DNase I protein expression in THP-1 cells, transfected or not with the DNase I-mCherry plasmid. β-Actin was used as a loading control. (B) Cellular localization of the DNase I protein by immunofluorescence after transfection of constructs in HeLa cells. Nuclei were stained using DAPI (blue), and mCherry constructions are in red. (C) Flow cytometry analysis of early apoptosis (annexin V^+^ 7-AAD^−^ cells) and late apoptosis/necrosis (annexin V^+^ 7-AAD^+^ cells) at 48 h posttransfection in HeLa cells. The error bars represent standard deviations (SD) from three independent experiments using a two-way analysis of variance (ANOVA). (D) *APOBEC3A* profiling by RT-qPCR at 24 h posttransfection of 250 ng of DNA and at 18 h after HSV-1 infection in THP-1 cells expressing DNase I or not and a negative control with a heat-inactivated (HI) virus. Data were normalized to the *RPL13A* housekeeping gene. Mean values and SEM were calculated for three independent experiments in duplicate (*n* = 6). (E) HSV-1 *UL42* expression by RT-qPCR at 18 h after HSV-1 infection (MOI of 0.2). Mean values and SEM were calculated for three independent experiments in duplicate (*n* = 6). ns, not statistically significant; ***, *P* < 0.005 (unpaired two-sided Student's *t* test).

To ensure that cytosolic DNA was the main inducer of *IFN* and *A3A* upregulation, a stable THP-1 cell line expressing DNase I-mCherry fusion protein was established by lentiviral transduction using cell sorting to select for mCherry expression. DNase I cleaves single- or double-stranded DNA, considerably reducing cytosolic DNA. Expression of DNase I-mCherry was checked by Western blotting, and the cellular localization was assessed by confocal microscopy (Fig. 2A and B). Early apoptosis (annexin V-positive 7-aminoactinomycin D [7-AAD]-negative cells) and late apoptosis/necrosis (annexin V^+^ 7-AAD^+^ cells) were analyzed and demonstrated that DNase I overexpression was not toxic for the cells (Fig. 2C).

The impact of the DNase I-mCherry construct was verified by transfecting dsDNA. When normalized to wild-type THP-1 cells, transfection of 250 ng of dsDNA generated an ∼2-fold decrease in *A3A* as well as *IFN-β* responses (Fig. 2D; Fig. S1D). THP-1 cells expressing DNase I were then infected with HSV-1 SC16 and compared to infected wild-type THP-1 cells. At 18 h postinfection, expression of *A3A* as well as *IFN-β* responses in DNase I-expressing THP-1 cells was ∼2-fold lower than in the wild-type cell line (Fig. 2D; Fig. S1D). To prove that the DNase I expressed in THP-1 was not involved in the degradation of viral DNA, reverse transcription-quantitative PCR (RT-qPCR) was performed on the HSV-1 *UL42* early gene (encoding the viral DNA polymerase processivity factor) and on two late genes, *UL44* (encoding the envelope glycoprotein C [gC]) and *US6* (encoding the envelope glycoprotein D [gD]). As observed in Fig. 2E and Fig. S2A, identical *UL42*, *UL44*, and *US6* mRNA expression was detected in the DNase I-expressing THP-1 cell line as well as in wild-type THP-1 cells, suggesting that viral replication was not influenced by DNase I.

10.1128/mBio.02557-21.2 FIG S2 HSV-1 and mitochondrial gene expression and mtDNA quantification. (A) HSV-1 *UL44* and *US6* late gene expression by RT-qPCR at 18 h after HSV-1 infection (MOI of 0.2) in THP-1 cells expressing or not expressing DNase I, upon KOS37 SPA or KOS37 UL98-SPA infections, upon EtBr treatment, or treated or not treated with ML-60218 at 25 μM. (B) mtDNA quantification by qPCR upon an infection with HSV-1 at an MOI of 0.2 in EtBr-treated cells or in THP-1 cells expressing DNase I or not. (C) HSV-1-infected cells were lysed and incubated with an anti-RIG-I or anti-IgG isotype monoclonal antibody to immunoprecipitated bound mitochondrial or viral RNA. SYBR RT-PCR quantification of mtRNA was performed using the mitochondrial genes *ND4, MT-CYTC*, and *MT-CYTB* and the viral genes *UL42* (early gene), *UL44* (late gene), and *US6* (late gene). Mean values and SEM were calculated for three independent experiments in duplicate (*n* = 6) (unpaired two-sided Student’s *t* test: **, *P* < 0.05; ***, *P* < 0.005; ns, not statistically significant). Download FIG S2, TIF file, 0.2 MB. Copyright © 2021 Berry et al. 2021 Berry et al. https://creativecommons.org/licenses/by/4.0/ This content is distributed under the terms of the Creative Commons Attribution 4.0 International license.

Together, these results proved that cytosolic DNA played a crucial role in the priming of the innate response. These findings highlighted a mechanism by which cytosolic DNA induces IFN-β, which in turn induces *A3A* expression.

### HSV-1 infection disturbs the mitochondrial network.

**FIG 3:**
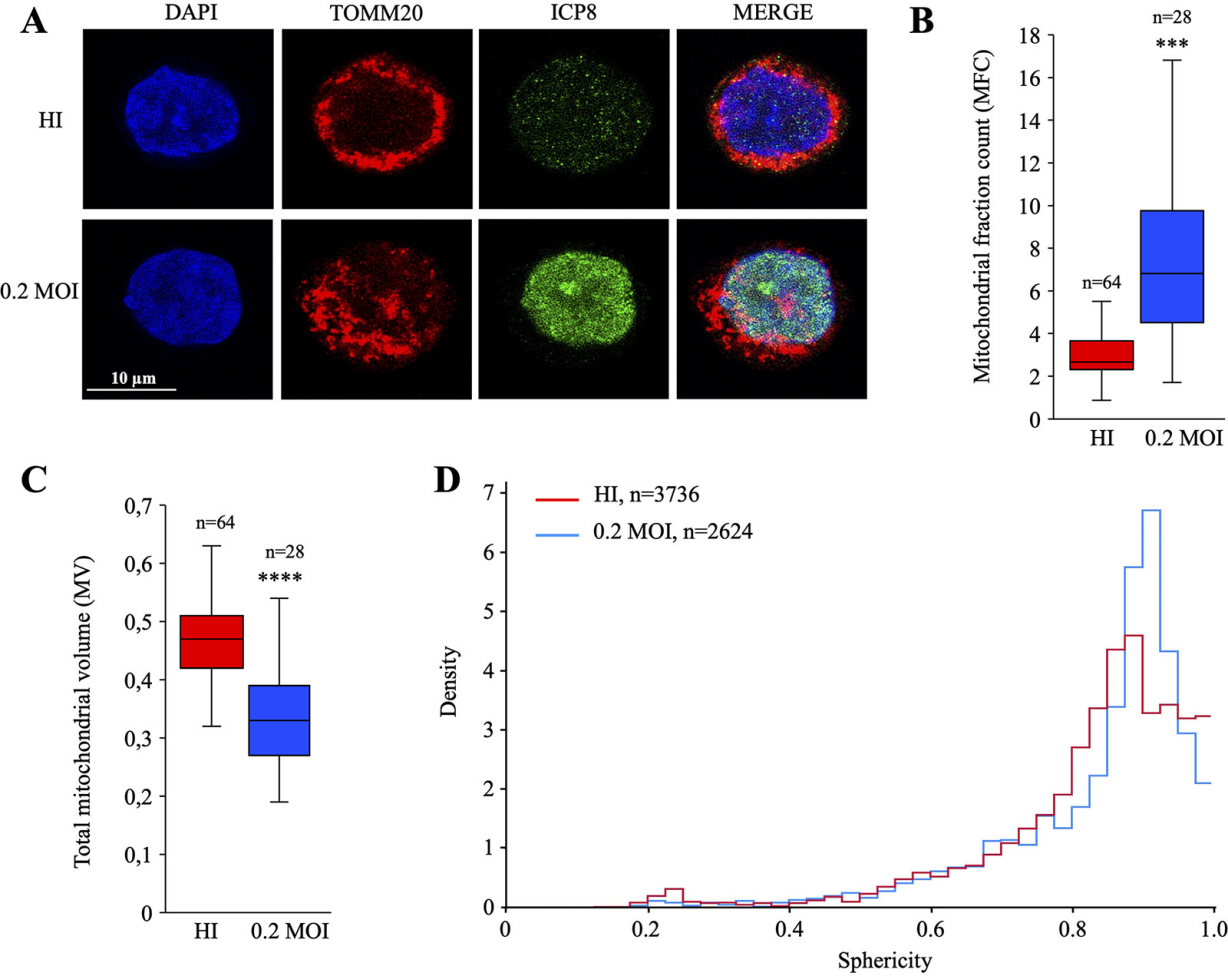
HSV-1 disrupted mitochondrial network. (A) Immunofluorescence of HSV-1-infected cells at 18 h postinfection by confocal microscopy. Nuclei were stained with DAPI (blue), HSV-1 particles with an anti-ICP8 monoclonal antibody (green), and mitochondria with an anti-TOMM20 monoclonal antibody (red). (B) Quantification of the mitochondrial fractional count (MFC). (C) Total mitochondrial volume (MV) normalized to the nucleus volume from confocal images. Box plots were statistically designed for 64 and 28 cells for HI (red) and HSV-1 at an MOI of 0.2 (blue), respectively. The *P* values in panels B and C are 10^−13^ and 10^−6^, respectively (Welch’s *t* test). (D) Evaluation of the sphericity of each mitochondrion; values describe an object’s closeness to a sphere when close to 1 and its opposite when close to 0. The quantity of mitochondria per sphericity value is represented by the density and was calculated for 3,736 and 2,624 mitochondria in cells infected with HI virus (red) and HSV-1 at an MOI of 0.2 (blue), respectively.

Loss of mitochondrial volume homeostasis and the accompanying mitochondrial swelling are two of the earliest and most striking signs of cell injury. Hence, in order to analyze different factors that are related to mitochondrial behavior following HSV-1 infection, THP-1 cells were infected with HSV-1 at an MOI of 0.2, and HI virus was used as a negative control. At 18 h postinfection, mitochondria and HSV-1 particles were stained with anti-TOMM20 (a mitochondrial import receptor subunit) and anti-ICP8 antibodies (Fig. 3A). Compared to the HI-virus-infected cells, the mitochondrial network of HSV-1-infected cells appeared to be disorganized and less aggregated around the nucleus. To statistically validate these observations, images acquired by confocal microscopy were deconvoluted and parameters related to the mitochondrial fraction count (MFC), total mitochondrial volume (MV) for the cell, and mitochondrial shape were analyzed. Twenty-eight infected cells and 64 noninfected randomly selected cells were imaged to calculate the respective MFCs. As observed in Fig. 3B, HSV-1-infected THP-1 cells demonstrated a more fragmented profile (mean MCF ≈ 7) than HI-virus-infected THP-1 cells (mean MCF ≈ 2.5), showing that HSV-1 infection led to ∼3 times more mitochondrial network fragmentation than in HI-virus-infected cells. Moreover, when calculating the mean of the total mitochondrial volume for infected and HI-virus-infected cells, we observed a significant decline in the average mitochondrial volume that was more pronounced in infected cells (0.47 versus 0.33) (Fig. 3C).

The density distribution of mitochondrial sphericity was analyzed for 3,736 and 2,624 mitochondria in HI-HSV-1-infected cells (Fig. 3D, red curve) and HSV-1-infected cells (Fig. 3D, blue curve), respectively. Values close to 0 correspond to considerably deformed mitochondria, with an abstract shape, whereas values close to 1 correspond to spherical mitochondria. As observed in Fig. 3D, the comparison of mitochondria in HI-virus (red curve) versus infected cells (blue curve) highlighted a more pronounced sphericity index in HSV-1-infected cells. All together, these data demonstrated that HSV-1 seemed to fragment and shape the mitochondrial network by reducing their volume, leading to more spherical mitochondria.

### HSV-1 UL12.5 promotes the release of mitochondrial DNA to the cytosol.

**FIG 4:**
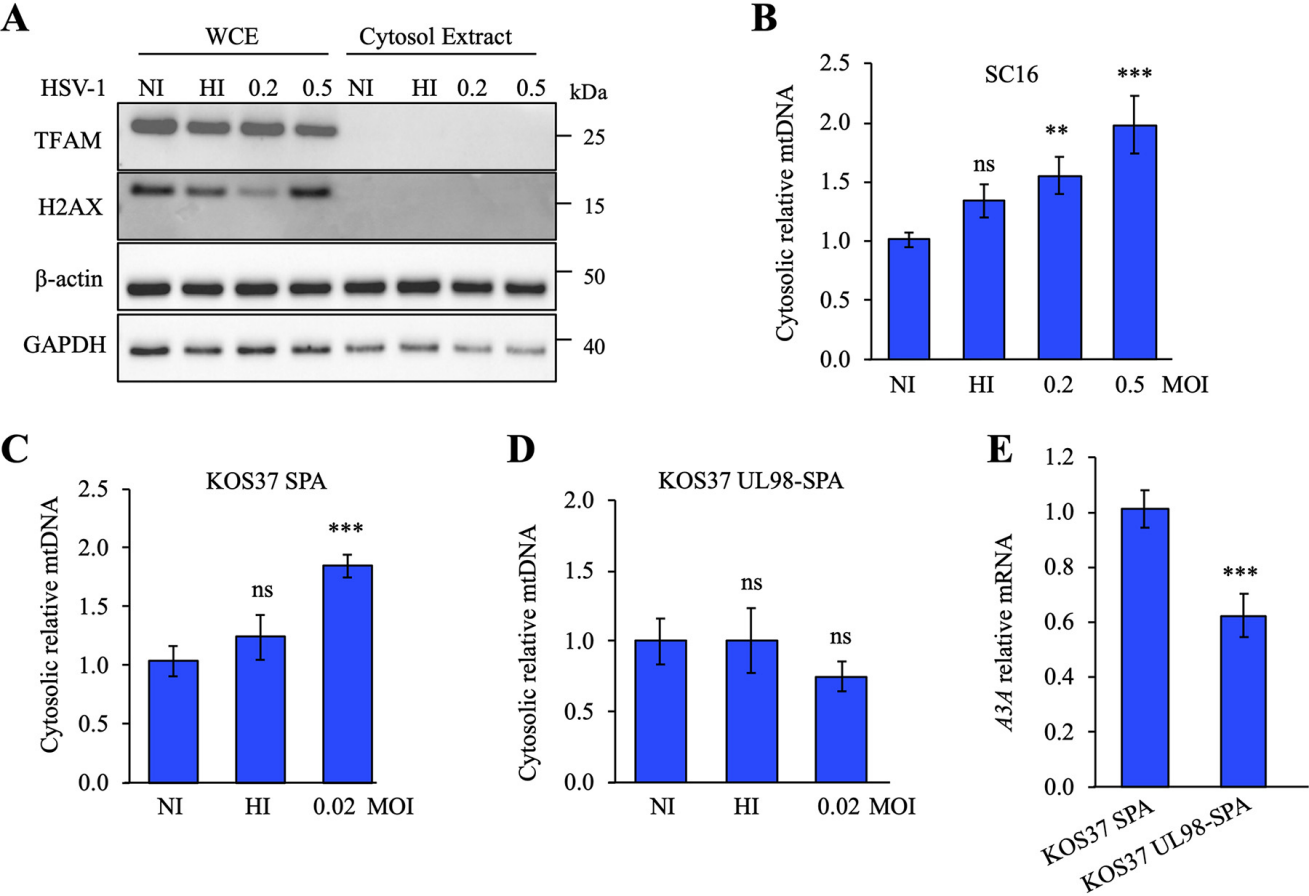
Cytosolic mitochondrial DNA release upon HSV-1 infection. (A) Purity control of cellular fractions by immunoblotting, using GAPDH to indicate the cytosol, TFAM for mitochondria, and H2AX as a marker of nuclear proteins; β-actin served as a loading control. WCE, whole-cell extracts; CE, cytosolic extracts. (B) THP-1 cells were fractioned at 12 h postinfection and mtDNA quantification was performed using the *MT-COI* gene in the cytosolic fraction and normalized to the quantification of the nuclear gene *β2M* of the total fraction. Mean values and SEM were calculated for three independent infections in duplicate (*n* = 6). (C and D) Cytosolic mtDNA qPCR was performed upon KOS37 SPA infection or upon KOS37 UL98-SPA infection using the *MT-COI* gene in the cytosolic fraction and normalized to the quantification of the nuclear gene *β2M* of the total fraction. (E) *A3A* relative expression upon KOS37 SPA or KOS37 UL98-SPA infection in THP-1 cells at 12 h postinfection. Gene expression was normalized using the expression level of the housekeeping gene *RPL13A*. Mean values and SEM were calculated for three independent infections in duplicate (*n* = 6). **, *P* < 0.05; ***, *P* < 0.005; ns, not statistically significant (unpaired two-sided Student's *t* test).

Having demonstrated that HSV-1 induced mitochondrial fragmentation, we next examined the involvement of cytosolic DNA sensors in stress signaling pathways. Hence, THP-1 cells were infected with HSV-1, and released mtDNA was quantified. At 12 h postinfection, THP-1 cells were subjected to digitonin fractionation, and whole-cell extracts (WCE) or cytosolic extracts (CE) were blotted using the indicated antibodies. As visualized in Fig. 4A, compared to β-actin and GAPDH, TFAM (mitochondrial transcription factor A) and histone H2AX, specific markers for mitochondria and nuclei, respectively, were missing from the cytosol extraction, demonstrating that the purification of the cytosol was not contaminated by remaining mitochondrial or nuclear proteins.

Quantification of mtDNA was performed by qPCR using the *MT-COI* (mitochondrial cytochrome *c* oxidase subunit I) gene present in the cytosolic fraction and normalized to the quantification of the nuclear gene *β2M* of the total fraction. Following HSV-1 infection, mtDNA in cytosolic extracts was increased in a dose-dependent manner by up to 2-fold (Fig. 4B), indicating a release of mtDNA. As the UL12.5 protein encoded by HSV-1 localizes to mitochondria and promotes rapid mtDNA depletion (8, 35), we investigated its impact in promoting *IFN-β* and *A3A* expression in uninfected cells. *UL12.5* and *UL98* (encoding a viral alkaline exonuclease homolog of UL12.5, in strain KOS37 UL98-SPA) were cloned in a pFLAP-GFP lentivirus-expressing vector, transfected into HEK-293T cells in order to produce lentiviral particles, and transduced in THP-1 cells. At 18 h posttransduction, Western blot analysis showed that UL12.5 and UL98 were produced similarly in HEK-293T and THP-1 cells (Fig. S3A). Confocal microscopy demonstrated a strong and significant colocalization of only UL12.5 with TOMM20 (Fig. S3B). Compared to one another, pFLAP-GFP (negative control)-, pFLAP-UL98-, and pFLAP-UL12.5-transduced THP-1 cells exhibited no significant increase of *IFN-β* (Fig. S3C), suggesting that UL12.5 probably needs viral and/or cellular cofactors to induce *IFN-β* and *A3A* expressions.

10.1128/mBio.02557-21.3 FIG S3 UL12.5 alone was not sufficient to promote *IFN-β* and *A3A* expression. (A) Western blot detection of SPA tagged UL98 or UL12.5 in HEK-293T and THP-1. β-Actin was used as a loading control. (B) Immunofluorescence detection in pFLAP-GFP, pFLAP-UL98, and pFLAP-UL12.5 THP-1 cell lines. Nuclei were stained with DAPI (blue), UL98 and UL12.5 with an anti-Flag (green) antibody, and mitochondria with an anti-TOMM20 monoclonal antibody (red). Confocal immunofluorescence demonstrated a strong and significant colocalization of only UL12.5 with TOMM20. (C) *IFN-β* profiling by RT-qPCR on the whole-cell extract, normalized using the expression level of the *RPL13A* housekeeping gene. Download FIG S3, TIF file, 0.5 MB. Copyright © 2021 Berry et al. 2021 Berry et al. https://creativecommons.org/licenses/by/4.0/ This content is distributed under the terms of the Creative Commons Attribution 4.0 International license.

To explore the effect of UL12.5 in an infectious context, we used a recombinant *UL12*-deficient HSV-1 strain which was complemented by HCMV *UL98*, which impaired its ability to induce mtDNA stress but was still able to replicate similarly to the parental strain (KOS37 SPA) (36).

THP-1 cells were infected with either KOS37 SPA or KOS37 UL98-SPA at an MOI of 0.02. The difference in MOI compared to the HSV-1 SC16 strain is due to the fact that the HSV-1 KOS37 strain has a more pronounced lytic effect in our experimental setup (Fig. 4B versus Fig. 4C and D). At 12 h postinfection, THP-1 cells were subjected to digitonin fractionation. WCE and CE were blotted and confirmed the absence of mitochondrial or nuclear contamination in the cytosol for both viral conditions (KOS37 SPA and KOS37 UL98-SPA) (Fig. S4A). Quantification of mtDNA demonstrated an ∼2-fold increase of cytosolic mtDNA with KOS37 SPA but failed to induce mtDNA release with KOS37 UL98-SPA (Fig. 4C and D). Infection with KOS37 UL98-SPA resulted in ∼1.7-fold attenuation of *A3A* and *IFN-β* expression (Fig. 4E; Fig. S4B), despite comparable *UL12*, *UL12.5*, and *UL98* HSV-1 protein expression (Fig. S4C) and *UL42*, *UL44*, and *US6* mRNA expression (Fig. S2A and S4D) and consistent with impaired antiviral innate immunity. All together, these data demonstrated that HSV-1 KOS37 SPA, expressing a functional UL12.5, was involved in the release of mtDNA in the cytosol that elicits a robust innate antiviral immune response.

10.1128/mBio.02557-21.4 FIG S4 THP-1 cells were infected with KOS37 SPA or KOS37 UL98-SPA (0.02 MOI). (A). At 12 h postinfection, THP-1 cells were fractioned and the purity was controlled by immunoblotting, using GAPDH to indicate the cytosol, TFAM for mitochondria, and H2AX as a marker of nuclear proteins. β-Actin served as a loading control. WCE, whole-cell extracts; CE, cytosolic extracts. (B) *IFN-β* relative expression upon KOS37 SPA or KOS37 UL98-SPA infections in THP-1 cells at 12 h postinfection. Gene expression was normalized using the expression level of the housekeeping gene *RPL13A*. Mean values and SEM were calculated for three independent infections in duplicate (*n* = 6) (unpaired two-sided Student’s *t* test: ***, *P* < 0.005). (C) Western blot analysis of UL12, UL12.5, and UL98 expression upon KOS37 SPA and KOS37 UL98-SPA infections. β-Actin protein expression was used as a loading control. NI, noninfected. (D) Viral *UL42* quantification by RT-qPCR upon KOS37 SPA and KOS UL98-SPA infections in THP-1 cells. Download FIG S4, TIF file, 0.3 MB. Copyright © 2021 Berry et al. 2021 Berry et al. https://creativecommons.org/licenses/by/4.0/ This content is distributed under the terms of the Creative Commons Attribution 4.0 International license.

### Cytosolic mtDNA is involved in the innate immune signaling pathways.

**FIG 5:**
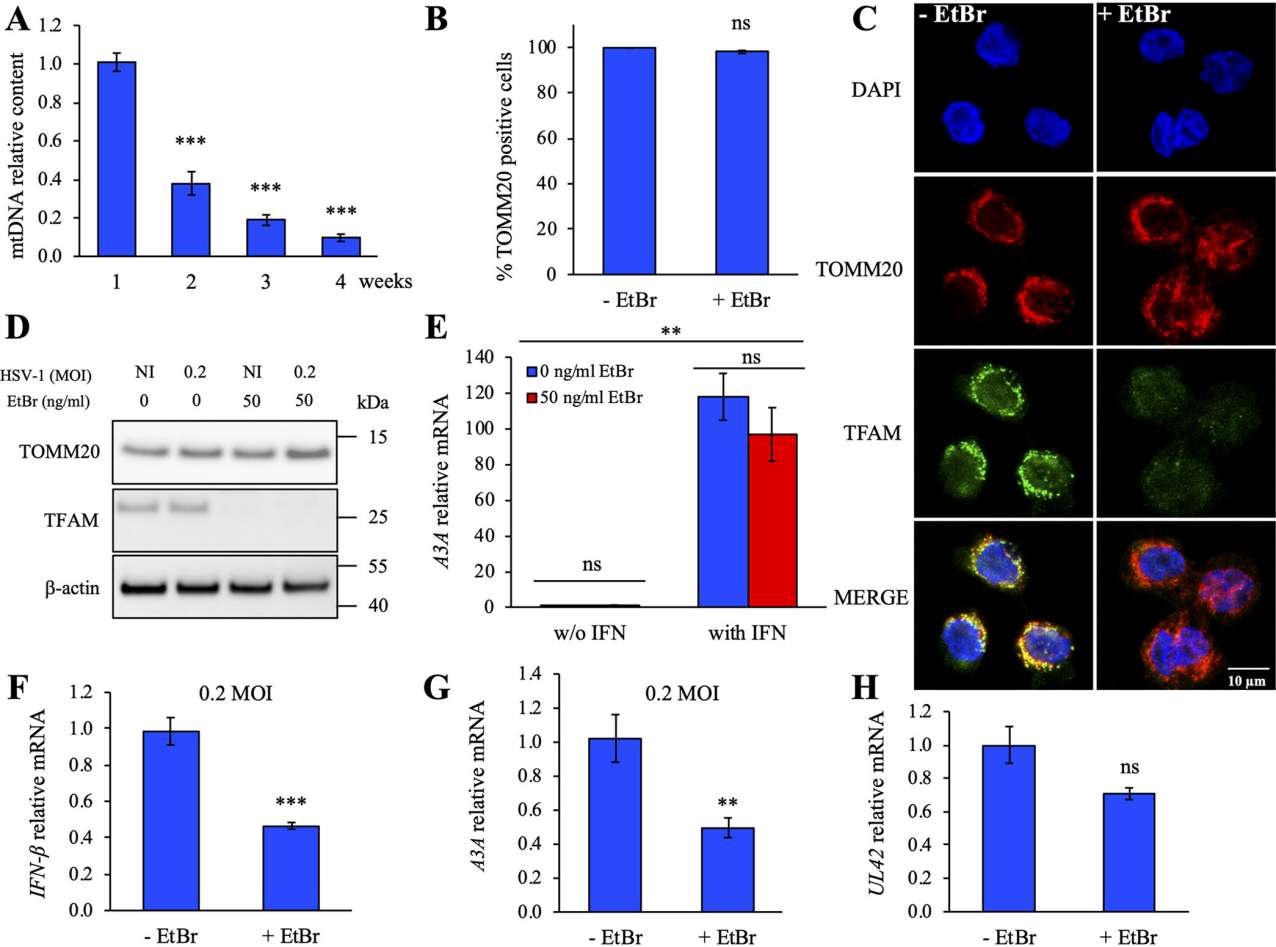
EtBr-treated THP-1 cells are depleted of mtDNA and underexpress the *A3A* gene. THP-1 cells were treated with 50 ng/mL of EtBr for 4 weeks. (A) Quantification of the mtDNA content upon EtBr treatment by qPCR using primers mt3319 and mt3212 (59). Data were normalized to the quantification of the nuclear gene *β2M*. Mean values and SEM were calculated for three independent cell lines treated with EtBr. Differences relative to nontreated cells were estimated using an unpaired two-sides Student's *t* test (**, *P* < 0.05). (B) Flow cytometry analysis of the TOMM20-positive cell population as a marker of the mitochondria mass in THP-1 cell lines, after 4 weeks of EtBr treatment. ns, not significant. (C) Immunofluorescence of EtBr-treated and nontreated THP-1 cell lines. Depletion of mtDNA was visualized by confocal microscopy. Nuclei were stained with DAPI (blue), mtDNA with an anti-TFAM monoclonal antibody (green), and mitochondria with an anti-TOMM20 monoclonal antibody (red). (D) Control of mtDNA depletion by immunoblotting with anti-TOMM20 and anti-TFAM antibodies against mitochondria and mtDNA, respectively. β-Actin was used as loading control. (E) Relative expression of *A3A* following IFN stimulation of THP-1 cell lines upon EtBr treatment or not, normalized to *RPL13A* housekeeping gene expression. (F and G) *IFN-β* and *A3A* profiling in EtBr-treated and nontreated THP-1 cells upon HSV-1 infection at an MOI 0.2 at 18 h postinfection. Data were normalized to *HPRT1* housekeeping gene expression. Mean values and SEM were calculated for three independent experiments in duplicate (unpaired two-sided Student's *t* test: **, *P* < 0.05; ***, *P* < 0.005; ns, not statistically significant). (H) Viral *UL42* quantification by RT-qPCR at 18 h after HSV-1 infection (0.2 MOI). Mean values and SEM were calculated for three independent experiments in duplicate (*n* = 6) (unpaired two-sided Student's *t* test: ns, not statistically significant).

To explore the consequences of mtDNA release to the cytosol, THP-1 cells were specifically depleted of mtDNA by ethidium bromide (EtBr) treatment (37–39). As can be seen in Fig. 5A, 50 ng/mL EtBr treatment caused a strong decrease of the mtDNA content that was proportional to the duration of treatment. Treatment of THP-1 cells with 50 ng/mL EtBr for 4 weeks resulted in ∼50% reduction of total mtDNA without loss of global mitochondrial mass, quantified by flow cytometry using TOMM20 staining (Fig. 5B; Fig. S2B). Furthermore, DNase I-expressing cells degrade ∼60% of the cytosolic mtDNA (Fig. S2B). There is evidence of residual mtDNA persisting following EtBr treatment or in DNase I-expressing cells.

Mitochondrial DNA and mitochondria were simultaneously stained with anti-TFAM and anti-TOMM20 antibodies, respectively. TFAM proteins are involved in the packaging of the mtDNA to form nucleoids, leading to a positive correlation between the proportion of TFAM proteins and the pool of mtDNA (40), whereas TOMM20 proteins belong to the outer membrane of the mitochondria. As observed in Fig. 5B and C, a strong reduction of the TFAM signal in the EtBr-treated cells was evident, while no difference was observed for the mitochondrial mass represented by the TOMM20 signal. As a control, protein samples extracted from HSV-1 infection in EtBr-treated and nontreated THP-1 cells were analyzed by Western blot and confirmed a massive depletion of mtDNA in EtBr-treated cells (Fig. 5D).

To prove that innate immune signaling pathways were not impaired in EtBr-treated THP-1 cells, IFN responsiveness was tested by quantifying *A3A* expression. As observed in Fig. 5E, EtBr-treated or nontreated cells incubated with IFN-supplemented medium for 24 h strongly expressed *A3A* ∼100× over background in the same manner, demonstrating that the innate immune signaling pathways were not impacted by the EtBr treatment. EtBr-treated and nontreated THP-1 cells were then infected with HSV-1 SC16. At 18 h postinfection, *IFN-β* and *A3A* gene expression was quantified. As can be seen in Fig. 5F and G, *IFN-β* and *A3A* levels were ∼2 times reduced in EtBr-treated THP-1 cells, demonstrating the involvement of mtDNA in triggering the innate immune signaling pathways upon HSV-1 infection. To prove that virus replication and gene expression were not inhibited in EtBr-treated cells relative to nontreated THP-1 cells, HSV-1 *UL42*, *UL44*, and *US6* mRNAs were amplified by RT-qPCR. As observed in Fig. 5H and Fig. S2A, *UL42*, *UL44*, and *US6* viral genes were similarly expressed in EtBr-treated and nontreated THP-1 cells, demonstrating that EtBr treatment does not impair viral replication.

### Cytosolic dsDNA is transcribed by RNA polymerase III and impacts RIG-I.

**FIG 6:**
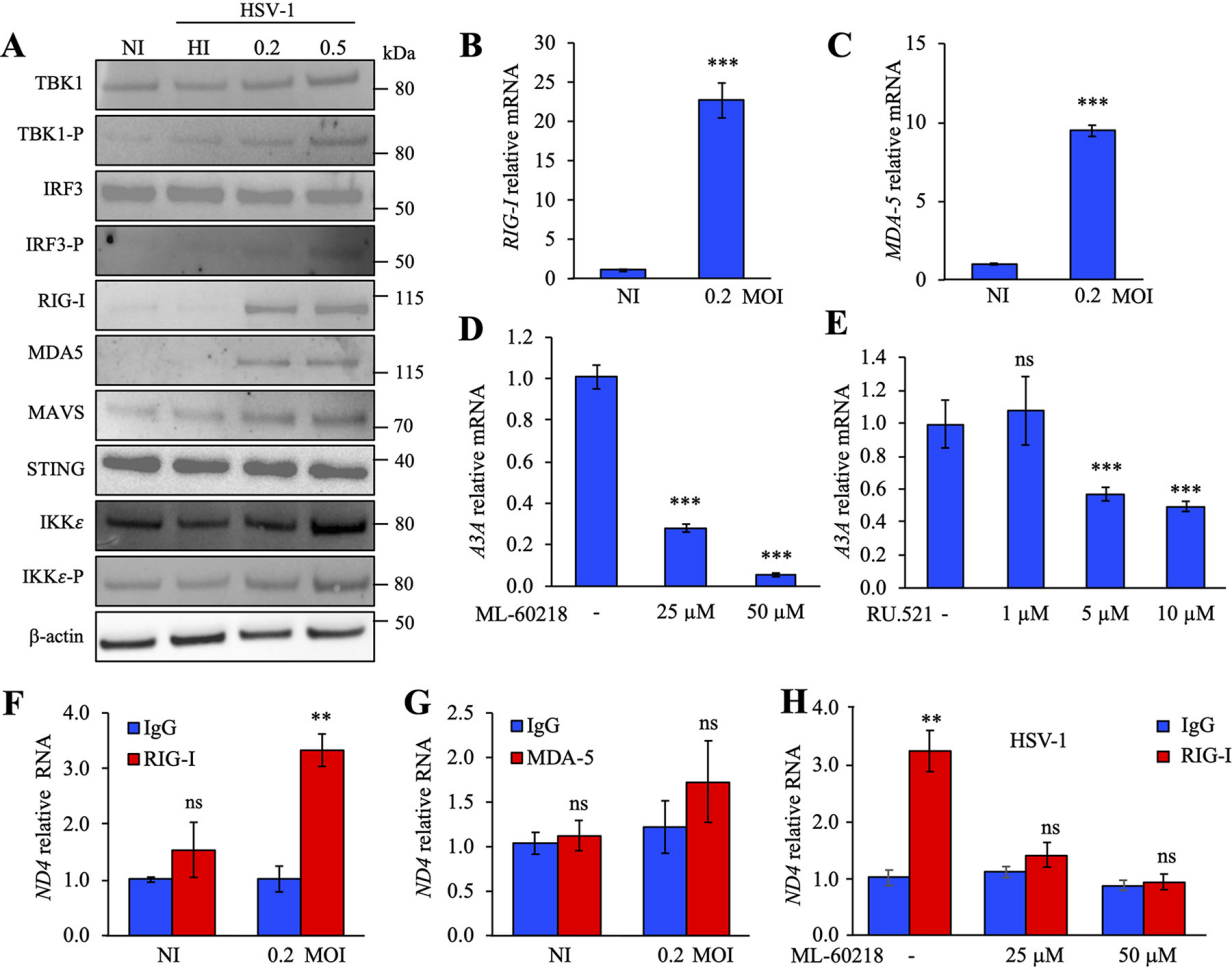
HSV-1 induced the RIG-I pathway via RNA polymerase III. (A) Western blot analysis of TBK1, TBK1-P, IRF3, IRF3-P, RIG-I, MAD5, MAVS, STING, IKKε, and IKKε-P at 18 h postinfection by HSV-1 SC16. β-Actin was used as a loading control. (B and C) Gene profiling of the IFN pathway after infection by HSV-1 at 18 h postinfection. *RIG-I* (B) and *MDA-5* (C) expression was normalized to *RPL13A* housekeeping gene expression. Mean values and SEM were calculated for three independent experiments in duplicate (*n* = 6). (D and E) *A3A* relative expression, normalized to *RPL13A* housekeeping gene expression, upon HSV-1 infection (MOI of 0.2) and concomitant treatment with ML-60218 (inhibitor of RNA polymerase III) at 25 μM or 50 μM or with RU.521 (inhibitor of cGAS sensor) at 1 μM, 5 μM, or 10 μM. (F and G) HSV-1-infected cells were lysed and incubated with an anti-RIG-I or anti-MDA5 monoclonal antibody along with an anti-IgG isotype used as a negative control to immunoprecipitate bound mtRNA. SYBR RT-PCR quantification of mtRNA was performed using the mitochondrial gene *ND4*. (H) THP-1 cells were infected with HSV-1 (MOI of 0.2), treated with ML-60218 at 25 μM and 50 μM, lysed, and incubated with an anti-RIG-I or anti-IgG monoclonal antibody. SYBR RT-PCR quantification of mtRNA was performed using the mitochondrial gene *ND4*. **, *P* < 0.05; ***, *P* < 0.005; ns, not statistically significant (unpaired two-sided Student's *t* test).

To identify the sensing molecules involved, THP-1 cells were infected at an MOI of 0.2. At 18 h postinfection, Western blot analysis showed increased levels of the phosphorylated forms of TBK1 (TBK1-P), IRF3 (IRF3-P), and IKKε (IKKε-P) as well as MOI-dependent increases in RIG-I, MDA5, and MAVS (Fig. 6A). Steady-state levels of STING and IKKε were unchanged. As observed, dose-dependent increases of TBK1-P and IRF3-P, key regulators of IFN production, suggest a signaling pathway via STING and/or MAVS. RT-qPCR performed on total extracted RNA from HSV-1-infected THP-1 cells demonstrated an upregulation of RIG-I and MDA5 expression ∼22 times and 10 times over background levels (Fig. 6B and C). As the RNA polymerase III and cGAS DNA sensor molecules are upstream of STING, we investigated in detail these two pathways.

Expression of the DNA sensors RNA polymerase III and cGAS, as well as STING, was not affected by HSV-1 infection (Fig. S5A to C). To discriminate between the two DNA sensors, THP-1 cells were infected with HSV-1 at an MOI of 0.2 along with ML-60218, an inhibitor of RNA polymerase III or RU.521, a cGAS pathway inhibitor (41). When cells were incubated with 25 μM and 50 μM ML-60218, a strong extinction of TBK1 and IRF3 phosphorylated forms was observed (Fig. S5D), leading to a dose-dependent decrease in *A3A* as well as in *IFN-β* expression (Fig. 6D; Fig. S5E). To prove that ML-60218 treatment does not impair viral replication in THP-1 cells, early (annexin V^+^ 7-AAD^−^ cells) and late (annexin V^+^ 7-AAD^+^) apoptosis/necrosis as well as HSV-1 *UL42*, *UL44*, and *US6* mRNA expression were analyzed. As visualized in Fig. S2A and F and S6A, THP-1 cells treated with ML-60218 did not induce apoptosis or impair viral replication. In contrast, when RU.521 was used, a less pronounced inhibition of *A3A* expression, ∼50%, was detected (Fig. 6E). To prove that the cGAS pathway was functional, THP-1 cells were incubated with 500 ng of G3-YSD, an agonist of the cGAS pathway, in the presence of 5 μΜ or 10 μM RU.521. As observed in Fig. S6B, addition of G3-YSD led to a strong expression of *A3A*, which is inhibited in the presence of RU.521, demonstrating that the cGAS pathway is functional.

10.1128/mBio.02557-21.5 FIG S5 Gene profiling of the interferon pathway after infection by HSV-1 at 18 h postinfection for genes for RNA polymerase III (A), cGAS (B), and STING (C). Expression was normalized to *RPL13A* housekeeping gene expression. (D) Western blot analysis of TBK1, TBK1-P, IRF3, and IRF3-P at 18 h after infection with HSV-1 (MOI of 0.2) and concomitant treatment with ML-60218 at 25 or 50 μM. (E) Relative expression of the *IFN-β* gene in the presence of ML-60218 at 25 or 50 μM. Expression was normalized to *RPL13A* housekeeping gene expression. (F) Flow cytometry analysis of early apoptosis (annexin V^+^ 7-AAD^−^ cells) and late apoptosis/necrosis (annexin V^+^ 7-AAD^+^ cells) in the presence of ML-60218 at 25 or 50 μM. Etoposide (Etop.) was used as a positive control. The error bars represent the SD from three independent experiments using a two-way ANOVA. Download FIG S5, TIF file, 0.3 MB. Copyright © 2021 Berry et al. 2021 Berry et al. https://creativecommons.org/licenses/by/4.0/ This content is distributed under the terms of the Creative Commons Attribution 4.0 International license.

10.1128/mBio.02557-21.6 FIG S6 HSV-1 *UL42* profiling and *A3A* relative expression in the presence of ML-60218, G3-YSD, or RU.521 inhibitor. (A) HSV-1 *UL42* profiling by RT-qPCR at 18 h after HSV-1 infection (MOI of 0.2) in the presence of ML-60218 at 25 μM. Mean values and SEM were calculated for three independent experiments in duplicate (*n* = 6) (unpaired two-sided Student’s *t* test: ns, not statistically significant). (B) *A3A* relative expression upon transfection of 500 ng/mL of G3-YSD (cGAS agonist) and the addition of RU.521 inhibitor at final concentrations of 5 μM and 10 μM at 18 h posttransfection. Mean values and SEM were calculated for three independent experiments in duplicate (*n* = 6) (unpaired two-sided Student’s *t* test: ns, not statistically significant; ***, *P* < 0.005). Download FIG S6, TIF file, 0.1 MB. Copyright © 2021 Berry et al. 2021 Berry et al. https://creativecommons.org/licenses/by/4.0/ This content is distributed under the terms of the Creative Commons Attribution 4.0 International license.

To show that cytosolic RNA synthesis by DNA-dependent RNA polymerase III binds to RIG-I, HSV-1-infected cells were lysed and incubated with anti-RIG-I or anti-MDA5 monoclonal antibodies along with an anti-IgG isotype used as a negative control to immunoprecipitate bound RNA. Total RNA was extracted and treated with DNase, and SYBR RT-qPCR was performed on NADH-ubiquinone oxidoreductase chain 4 (*ND4*), cytochrome *c* (*MT-CYTC*) and cytochrome *b* (*MT-CYTB*) mitochondrial genes along with *UL42*, *UL44*, and *US6* viral genes. As observed in Fig. 6F and Fig. S2C, ∼3 to 4 times more *ND4*-, *MT-CYTC*-, and *MT-CYTB*-specific RT-PCR products were recovered only from the anti-RIG-I immunoprecipitates, which demonstrates that RIG-I remained the major sensing molecule. In comparison, RT-PCR products obtained from anti-MDA5 immunoprecipitates (Fig. 6G) or THP-1 cells treated with 25 μM and 50 μM ML-60218 (Fig. 6H) were not significantly different from the background. In addition, RIG-I bound HSV-1 viral RNA, compared to IgG, contained ∼2 times more *UL42*, *UL44*, and *US6* specific RT-PCR products (Fig. S2C). Together, these data demonstrated that upon HSV-1 infection, IFN was induced by cGAS and RNA polymerase III/RIG-I pathways and both mitochondrial and viral RNAs could bind to RIG-I, leading to IFN pathway induction, with, however, mtRNA producing a higher impact on IFN production.

### Analyses of cytosolic mtDNA mutations reveal an APOBEC3A signature.

To analyze the mutational state of cytosolic mtDNA, *MT-COI* amplicons were generated by PCR from NI, HI-virus-infected, or HSV-1-infected cells at MOIs of 0.02 or 0.2 and were analyzed by next-generation sequencing (NGS). For each sample, reads obtained by Illumina sequencing were checked for quality and assessed using FastQC. Reads were analyzed using BLAST and a homemade Python script. The base substitution mutational signatures were described using a classification based on the six classes of single-base mutations: C to A, C to G, C to T, T to A, T to C, and T to G. To provide greater depth of insight into the operative mutational processes, the sequence context in which mutations occurred was incorporated by considering the bases immediately 5′ and 3′ of each mutated base.

All trinucleotide motifs (based on the reference sequences) as well as nucleotide substitutions contained in all reads were counted for each sample. Mutation content was obtained by dividing the number of each substitution in a given context by the count of the related trinucleotides sequence (i.e., number of 5′XpCpN3′-to-5′XpTpN3′ mutations divided by 5′XpCpN3′ motif count, with X and N being random nucleotides). The four data sets (NI, HI virus infected, HSV-1 infected at an MOI of 0.02, and HSV-1 infected at an MOI of 0.2) contained between 796,846 and 1,033,932 reads. Approximately 68.5% of the reads did not contain any mutation. The proportion of mutations to trinucleotide motifs is ∼0.41% for each sample.

**FIG 7:**
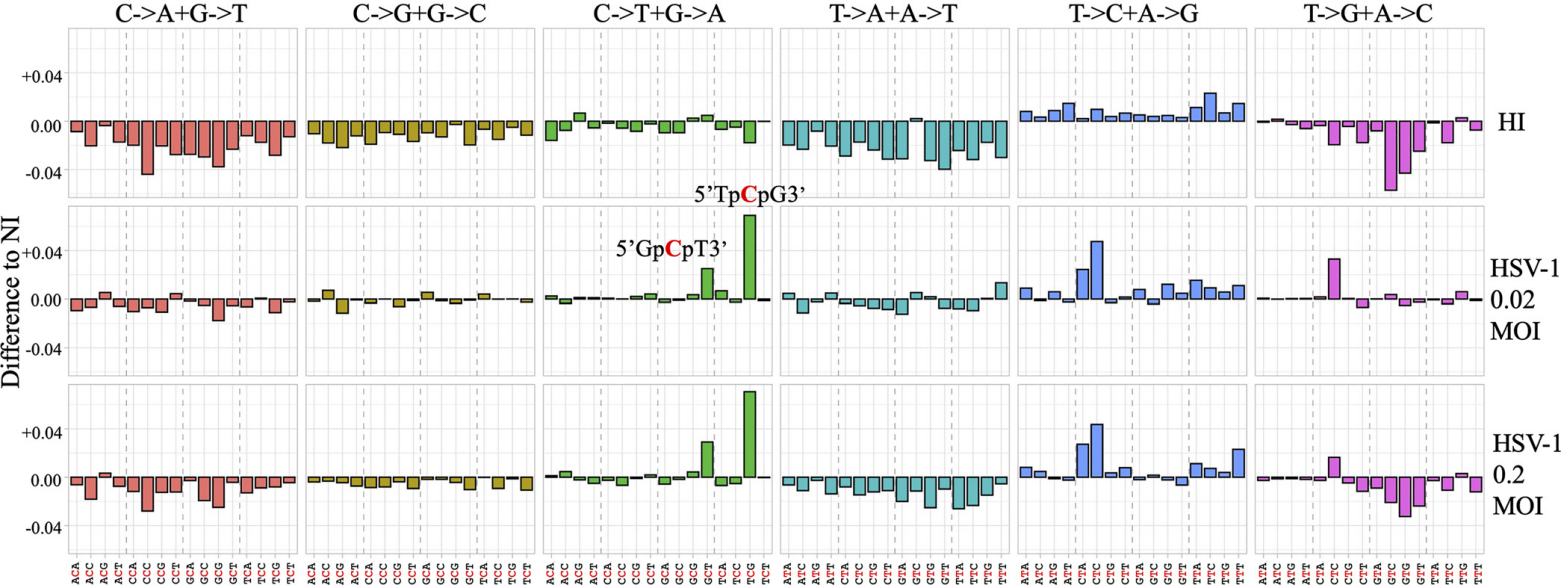
Pattern of mutations and contexts analyzed in mtDNA. The difference in mutation proportion between HSV-1 at MOI of 0.02 and 0.2 and the HI-virus-infected sample is shown.

Mutation content was analyzed for each sample based on substitutions with their respective contexts. To estimate the implication of HSV-1 infection in modulating the proportion of specific substitutions, the difference in mutation content observed between HI-virus-infected samples, samples infected with HSV-1 at an MOI of 0.02, samples infected with HSV-1 at an MOI of 0.2, and NI samples was investigated (Fig. 7). By comparing the differences between HSV-1-infected THP-1 cells, HI-virus-infected cells, and NI cells, we observed that C-to-T substitutions were predominant (Fig. 7). The most significant difference was observed for the 5′TpCpG-to-5′TpTpG substitution (the underlined nucleotide represents the mutated base) and slightly in the context 5′GpCpT to 5′GpTpT. The other substitutions fluctuated very slightly and had a difference lower than the greatest difference observed between NI and HI-virus-infected samples. We note that 5′TpCpG-to-5′TpTpG substitutions were not observed in a control sample generated from amplification of a plasmid following the identical protocol. Although 5′YpTpCpW has been described as a signature for A3A (42, 43), the preferred context we obtained was not without precedent; indeed, this specific signature is consistent with the mutagenic properties of A3A cytidine deaminase, which also preferentially targets 5′TpCpG motifs (33, 34).

## DISCUSSION

These data demonstrated that in a human cellular model, HSV-1 infection disrupts the mitochondrial network with a remodeling of mitochondrial shape, resulting in a fragmentation of the mitochondria associated with a decrease in their volume and an increase in their sphericity. These data reinforce the notion that mitochondrial morphology in infected HSV-1 cells is not a static event, as HSV-1 infection leads to a loss of mitochondrial motility by fragmentation of the mitochondria (44). Therefore, since HSV-1 infects many cell types, it would not be surprising if it induces differential modulations of mitochondrial morphology in order to regulate cellular functions related to mitochondrial function, such as apoptosis or innate immunity signaling (45, 46).

Despite a convergence of the different signaling pathways toward the same activation of the innate immune response, the mechanisms underlying the release of mtDNA into the cytosol differ according to the model used. Indeed, it was shown previously in a mouse model that HSV-1 infection causes hyperfusion of the mitochondrial network by the action of the UL12.5 viral exonuclease (10). Conversely, our results associated virus-induced mitochondrial stress with fragmentation of the mitochondria, leading to a reduction in volume and an increase in sphericity (Fig. 3). Our data are derived from experiments carried out in human cells, which could explain the fragmentation associated with mitochondrial damage. Interestingly this is not without precedent, as it has been demonstrated that infection of HaCaT immortalized keratinocytes with HSV-1 resulted in an intracytoplasmic relocation of the Drp1 protein to the outer membrane of the mitochondria, which is involved in fission mechanisms, leading to fragmentation of the mitochondrial network (47).

Mitochondrial morphological changes can be associated with mtDNA release in the cytosol, potentially involving the action of the HSV-1 UL12.5 viral exonuclease, which targets mitochondria through its mitochondrial localization sequence (8, 35). However, in contrast to the mouse model (10), UL12.5 expression alone in THP-1 cells was not able and sufficient on its own to stimulate IFN-β (Fig. S3C), suggesting that other cofactors are probably involved in the release of mtDNA, leading to the induction of *IFN-β* and *A3A*. Indeed, it has been shown that ICP8 could interact with UL12.5, probably interfering with its function (9).

It is now well documented that cytosolic DNA, whatever its origins, plays an essential role in the IFN response, triggering the innate immune signaling pathway. Indeed, infection of a THP-1 lineage stably overexpressing cytoplasmic DNase I showed that cytosolic DNA was the trigger for IFN signaling following viral infection (Fig. 2D; Fig. S1D). Although we cannot exclude direct sensing of HSV-1 DNA, we demonstrated the contribution of cytosolic mtDNA in triggering IFN signaling response. In the same way, we showed that HSV-1-infected THP-1 cells lacking mtDNA following EtBr treatment (Fig. 5) demonstrated ∼2-times-reduced expression of *IFN-β* and *A3A*, proving the involvement of mtDNA in triggering of the innate immune signaling pathway (Fig. 5F and G).

Which DNA-sensing pathway is involved during HSV-1 infection? A large number of cellular DNA sensors have been identified and characterized, which greatly broadened our knowledge of host-virus interaction. However, it has been shown that several DNA sensors exert distinct functions in different cell types or in response to different viruses. Among these cellular DNA sensors, cGAS can sense HSV-1 DNA and trigger host immune responses upon infection (48, 49). We demonstrated here that mtDNA released in the cytosol following HSV-1 infection was detected by RNA polymerase III (Fig. 6E). This is not without precedent; indeed, other viruses, such as adenovirus and Epstein-Barr virus, induce *IFN-β* expression in an RNA polymerase III-dependent manner through activation of RIG-I (11, 12). Moreover, it was recently demonstrated that HSV-1 infection leads to the relocalization and exposure of the cellular *5S* rRNA pseudogene 141 transcript, allowing its recognition by RIG-I (50).

A specific inhibitor used against RNA polymerase III (ML-60218) significantly reduced *IFN-β* responses and *A3A* expression (Fig. 6D; Fig. S5E). Interestingly, we detected a more pronounced inhibition of IFN-β/A3A production in infected HSV-1 cells in the presence of ML-60218 (Fig. 6D; Fig. S5E) than in KOS37 UL98-SPA-infected cells (Fig. 4E; Fig. S4B) or with infected cells treated by EtBr (Fig. 5F and G). Concentrations of ML-60218 used in the present work were not toxic for the cells (Fig. S5F) but probably have a stronger inhibitory effect on IFN-β/A3A production than KOS37 UL98-SPA or cells treated with EtBr. Obviously, other unidentified parameters could influence the release of mtDNA that leads to IFN-β/A3A production.

As already documented, we also proved by using specific inhibitors that cGAS is also involved in the induction of IFN-β/A3A (Fig. 6E). Transcription of cytosolic mtDNA by RNA polymerase III allowed the synthesis of cytosolic and viral RNA that binds RIG-I (Fig. 6F; Fig. S2C), leading to the activation of the IFN signaling pathway. It is also conceivable that depending on HSV-1 tropism, signaling pathways leading to the production of IFN could be different.

Interestingly, the absence of a functional RNA polymerase III has been documented in children with severe varicella-zoster virus (VZV) infection (51), a virus also belonging to the family *Herpesviridae*. This worsening of the clinical picture has been associated with a defect in the IFN response, related to a genetic deficiency of RNA polymerase III due to heterozygous mutations (51). This highlights the essential role of RNA polymerase III in the antiviral response to VZV infection.

To increase the chance of survival, some viruses appear to have adopted the strategy of damaging the host cell mitochondrial DNA. Since mitochondria function as a source of energy and play an important role in antiviral immunity, it is possible that damage to mitochondrial DNA may help in evading mitochondrial antiviral immune responses (1). Some viruses have found different ways to degrade cytosolic mtDNA. Indeed, dengue fever virus induces mitochondrial elongation through the viral protein NS4B, and mitochondrial hyperfusion appears to have a proviral role in reducing IFN response (52). In the same vein, influenza virus-encoded NS1 protein, which interacts with the cytosolic mtDNA, leads to a reduction in the induction of IFN (5). As A3 DNA mutators are downstream of IFN induction, another viral strategy could be to exclude A3 from replication and transcription sites. Recent studies reported the relocation of A3A from the nuclear to cytoplasmic compartment, as is the case during HSV-1 infection (53, 54). This was observed for a variety of viral proteins, in particular with HSV-1 and ICP6, EBV and BORF-2, and KSHV and ORF61 (53, 54).

As a consequence, A3A relocation in the cytoplasm will lead to an increase of C-to-T editing in cytosolic DNA. Indeed, by performing deep sequencing of cytosolic mtDNA, we observed an increase of C-to-T editing occurring mainly in a specific context, 5′TpCpG (Fig. 7). As A3B is exclusively located in the nucleus, this excludes mtDNA editing by this enzyme (55, 56). These characteristics are consistent with the mutagenic properties of A3A cytidine deaminase, which was described to target 5′TpCpG motifs in single-stranded DNA (33). On the other hand, the role of A3A and A3B in cancer was highlighted by the fact that many cancer genomes encode tens of thousands of C-to-T transitions frequently in the 5′TpCpA and 5′TpCpT dinucleotide context (30–32) and in 5′TpCpG, if the genomic underrepresentation of this motif is accounted for (33, 34). The difference between the signatures of nuclear and mitochondrial DNA could be linked to the affinity of A3A enzyme for the ssDNA substrate, which could be a function of substrate nucleotide composition, length, secondary structure, and pH variation (34).

The innate immune system is sensitive to dsDNA, with cellular sources of immune stimulatory self-DNA including viral DNA as well as nuclear DNA, cytosolic mtDNA, and DNA from phagosomal compartments. This suggests that foreign DNA, whatever its origin, present in the cytosol constitutes a danger signal and should be shut down by enzymatic processes, in order to reduce innate immune activation. In this sense, IFN activated *A3A* gene expression, which led to increased catabolism of ssDNA generated by cytoplasmic exonucleases (13, 24, 56). We also previously noticed that up to 17% of primary CD4^+^ T cells showed signs of A3-edited cytosolic mtDNA, demonstrating a very dynamic image of the mitochondrial network (55).

Together, these results demonstrate that viral stress induced by HSV-1 results in mitochondrial fragmentation. These changes in term of shape and sphericity help the release of mitochondrial DNA. Accumulation of mtDNA in the cytosol that probably escaped autophagic clearance will induce RNA polymerase III/RIG and cGAS pathways, leading to the expression of type I IFN and A3A cytidine deaminase. A3A could then probably lead to the catabolism of cytosolic mtDNA to decrease the danger signal. Finally, an HSV-1 infection, and probably any pathways involved in the induction of IFN, will inevitably lead to the presence of somatic mutations in cytosolic DNA and, to a lesser extent, mutations in nuclear DNA, assuming that the cell survives. As chronic inflammation underlies many cancers, while we live in an antigenic world, APOBEC3 mutator enzyme-induced somatic mutations are an inevitable part of human biology.

## MATERIALS AND METHODS

### Reagents.

RNA polymerase III inhibitor (ML-60218) was from Merck Millipore, and cGAS inhibitor (ihn-ru521) and cGAS agonist (G3-YSD; tlrl-ydna) were from InvivoGen. MDA5 (D74E4; rabbit monoclonal antibody [MAb] 5321), STING (antibody 3337), phospho-IRF-3 (Ser396) (4D4G; rabbit MAb 4947), IRF-3 (D6I4C; XP rabbit MAb 11904), RIG-I (D14G6; rabbit MAb 3743), MAVS (antibody 3993), TBK1/NAK (D1B4; rabbit MAb 3504), phospho-TBK1/NAK (Ser 172) (D52C2; rabbit MAb 5483), IKKε (D20G4; rabbit MAb 2905), phospho-IKKε (Ser172) (D1B7; rabbit MAb 8766), β-actin horseradish peroxidase (HRP) conjugate (13E5; rabbit MAb 5125), HRP-linked anti-rabbit IgG (antibody 7074), and histone H2AX (antibody 2595) antibodies were from Cell Signaling Technology. GAPDH (mouse MAb G8795) was from Sigma-Aldrich. Anti-mouse IgG HRP-linked (antibody NA931V) was from GE Healthcare. Monoclonal anti-β-actin−peroxidase (antibody A3854) and digitonin (D141) were from Sigma-Aldrich. Monoclonal anti-mouse TFAM (18G102B2E11) and Turbo DNase (2 U/μL; AM2238) were from Invitrogen. GlycoBlue coprecipitant (AM9515) was from Applied Biosystems. TOMM20 (EPR15581-39; rabbit MAb ab186734), ICP8 (11E2; mouse MAb ab20194), and mCherry (1C51; rabbit MAb ab125096) were from Abcam. RIG-I (D33H10; rabbit MAb 4200) and mouse anti-rabbit IgG (conformation specific; L27A9; MAb 3678), cell lysis buffer (9803), protein A magnetic beads (73778), glycine (7005), and phenylmethylsulfonyl fluoride (PMSF) (8553) were from Cell Signaling. Recombinant RNasin RNase inhibitor (N2515) was from Promega. Secondary Alexa Fluor 488 goat anti-mouse immunoglobulin (A11029), Alexa Fluor 488 goat anti-rabbit immunoglobulin (A11034), Alexa Fluor 633 goat anti-mouse immunoglobulin (A21053), Alexa Fluor 647 goat anti-rabbit immunoglobulin (A21244), Fluoromount-G with DAPI (4′,6-diamidino-2-phenylindole; 00-4959-52), and SlowFade Diamond antifade mountant without DAPI (S36972) were from Invitrogen. DAPI solution 1.0 mg/mL (564907) was from BD Pharmingen. Monoclonal Alexa Fluor 647 mouse anti-H2AX (pS139) (560447) was from BD Pharmingen. The annexin V apoptosis detection kit (888007) with annexin V eFluor 450 (48-8006-69) and the fixable viability dye eFluor 780 (65-0865-14) were from eBioscience. CD14 MicroBeads (human; 130-050-201) were from Miltenyi Biotec. JetPRIME (114-07) was from Polyplus Transfection, and Lipofectamine RNAiMAX (13778075) was from Invitrogen. Sodium pyruvate (100 mM; 11360070) and HEPES buffer (1 M; 15630) were from Gibco. Uridine (U3003), glucose solution (100 g/L; G8644), and ethidium bromide solution (10 mg/mL in H_2_O; E1510) were from Sigma. NucleoSpin gel and the PCR clean-up kit (740609) were from Macherey-Nagel. The Quant-iT dsDNA assay kit (high sensitivity; Q33120) was from Invitrogen, and the NEBNext DNA library preparation kit (E7645) and NEBNext multiplex oligonucleotides for Illumina (E7600) were from New England Biolabs.

### Cell lines.

THP-1 cells (ATCC TIB-202) were maintained in Roswell Park Memorial Institute medium (RPMI 1640; Gibco), supplemented with heat-inactivated fetal calf serum (10%), penicillin (50 U/mL), streptomycin (50 μg/mL) and β-mercaptoethanol (0.05 mM) were grown in 150-cm^2^ cell culture flasks. Human HeLa cells, Vero cells, and HEK-293T cells were maintained in Dulbecco’s modified Eagle’s medium (DMEM; Gibco), supplemented with heat-inactivated fetal calf serum (10%), penicillin (50 U/mL), and streptomycin (50 μg/mL) and were grown in 75-cm^2^ cell culture flasks in a humidified atmosphere containing 5% CO_2_.

### Construction of THP-1 DNase wild-type cell line.

From the lab collection, a plasmid harboring the sequence of the cDNA of the wild-type DNase I gene, previously designed by GeneCust, was cloned in pcDNA3.1/V5 His-TOPO under the control of the CMV promoter, and the mCherry plasmid was cloned in pCR2.1 vector. Both were digested and ligated together. Ligated plasmid pcDNA 3.1 DNase (WT)-mCherry was used to insert the sequence of interest inside the lentiviral vector pTRIP harboring the promoter EF1α. HEK-293T cells were used to produce lentiviral vectors upon Fugene HD (Promega) transfection of DNase-mCherry vectors along with a p8.74 packaging plasmid and a VSV-G envelope-encoding plasmid. Then, THP-1 cells were transduced with lentiviral vectors purified from HEK-293T supernatants. mCherry-positive cells were sorted and amplified for establishing of stable cell lines.

### Construction of THP-1 cell lines expressing UL98 and UL12.5.

UL98-SPA and UL12.5-SPA sequences, encoding viral proteins C-terminally tagged with a sequential peptide affinity (SPA) tag were PCR amplified using Q5 high-fidelity DNA polymerase (New England Biolabs) from plasmids obtained from James R. Smiley (57). Amplifications were performed with forward primers designed to add a 5′ BamHI restriction site (UL12.5-BamH1fwd and UL98-BamH1fwd) and a reverse primer containing a SGRD1 restriction site 3′ of the SPA tag coding sequence, SPA-SGRD1rev (Table S1). Amplicons were subcloned into the TOPO 2.1 vector (Life Technologies), and the resulting plasmids were digested using BamHI/SGRD1 restriction enzymes and cloned into the pFLAP-EF1α-GFP-IRES-GFP lentiviral vector. Lentiviral vectors were produced from HEK-293T cells transfected using a standard calcium chloride protocol, with a p8.74 packaging plasmid, a VSV-G envelope-coding plasmid, and either pFLAP-EF1α-GFP-IRES-GFP, pFLAP-EF1α-UL98-IRES-GFP, or pFLAP-EF1α-UL12.5-IRES-GFP. Supernatants were collected, microfiltered through a 0.45-μm-pore-size sterile filter unit (Stericup; Millipore), and concentrated by ultracentrifugation for 90 min (26,000 rpm) through a 20% sucrose–phosphate-buffered saline (PBS) cushion. Lentiviral pellets were resuspended in PBS and used to transduce THP1 cells in the presence of Polybrene (5 μg/mL; Sigma). After 72 h, GFP-positive cells were sorted and amplified to generate stable cell lines.

10.1128/mBio.02557-21.7 TABLE S1 Compendium of primers used for PCR, qPCR, and RT-PCR; qPCR was done with either SYBR green or TaqMan with the UPL probes listed. Fwd: forward, rev: reverse. Download Table S1, DOCX file, 0.02 MB. Copyright © 2021 Berry et al. 2021 Berry et al. https://creativecommons.org/licenses/by/4.0/ This content is distributed under the terms of the Creative Commons Attribution 4.0 International license.

### Preparation of mtDNA-depleted cell lines.

THP-1 cells were treated with 50 ng/mL of ethidium bromide (Sigma) for up to 4 weeks to generate cell lines depleted of mtDNA. EtBr-treated and nontreated THP-1 (control) cells were maintained in RPMI supplemented with heat-inactivated fetal calf serum, penicillin, streptomycin, and β-mercaptoethanol and specially complemented with sodium pyruvate (1 mM; Gibco), uridine (50 μg/mL; Sigma) and glucose at a final concentration of 4.5 g/L (Sigma).

### PBMC purification.

Blood from healthy patients was provided by the Office of Biological Products of the Institut Pasteur. PBMC purification based on the Ficoll method (Ficoll-Paque Plus; GE Healthcare) was performed according to the guidelines of MACS Miltenyi Biotec. Primary monocytes were isolated using CD14 microbeads (Miltenyi Biotec) and maintained in supplemented RPMI for 3 days.

### Virus strain and plaque assay.

The wild-type HSV-1 strain SC16 was kindly provided by Marc Labetoulle (I2BC, Gif-sur-Yvette, France). James R. Smiley (University of Alberta, Canada) kindly provided the KOS37 SPA and KOS37 UL98-SPA strains. All HSV-1 stocks were prepared as previously described (58). For the virus titration by plaque assay, 2 million Vero cells were grown in 6-well plates. After 24 h, cells were infected with several dilutions (10^−2^ to 10^−6^) of HSV-1 in medium without serum. After 1 h, the inoculum was removed to avoid secondary infection and wells were covered with a solution of Avicel (2.4%)-complete DMEM. After 72 h at 37°C, the solution of Avicel was removed, and cells were fixed with 4% paraformaldehyde after washing. Plaques were stained with crystal violet (2.3%) and counted manually.

### HSV-1 infection.

THP-1 cells or primary monocytes were infected with HSV-1 at a multiplicity of infection (MOI) of 0.1, 0.2, 0.5, 1, or 3 for 18 h. As a negative control, cells were infected at an MOI of 0.5 with HSV-1 that had been inactivated at 70°C for 30 min. For both infections, viruses were absorbed in serum-free RPMI for a defined period at 37°C before dilution with complete RPMI. To study the role of the RNA polymerase III or cGAS, 2 h postinfection, inhibitors were added in the culture medium at final concentrations of 25 and 50 μM for ML-60218 (Merck Millipore) or at final concentrations of 1, 5, and 10 μM for RU.521 (InvivoGen).

### THP-1 stimulation.

DNase cells were assayed for their ability to degrade DNA, compared to wild-type THP-1 cells, by transfection of 250 ng of DNA with JetPRIME (Polyplus Transfection). EtBr-treated and nontreated THP-1 cell lines were tested for their ability to respond to IFN stimulation by addition of IFN-supplemented supernatant to the culture medium for 24 h.

The ability of RU.521 (InvivoGen) to inhibit the cGAS pathway, as well as the expression of IFN and APOBEC3A, was tested by incubating THP-1 cells with the inhibitor at final concentrations of 5 μM and 10 μM for 1 h before the transfection of 500 ng of G3-YSD (InvivoGen) using Lipofectamine RNAiMAX (Invitrogen).

### Subcellular fractionation.

Ten million THP-1 cells were infected with HSV-1. At 12 h postinfection, cells were recovered and separated into equal samples. One sample was considered the whole-cell extract (WCE). The second sample was resuspended in digitonin buffer (150 mM NaCl, 50 mM HEPES [pH 7.4], and 25 μg/mL of digitonin) and other aliquots were kept for future RNA or protein analysis. After 10 min of incubation with gentle agitation, lysate was centrifuged for 3 min at 980 × *g* to collect nuclei and unbroken cells. Centrifugation was repeated three times. Supernatant was centrifuged at 10,000 × *g* for 10 min to collect mitochondria. Finally, supernatant was centrifuged at 20,000 × *g* for 30 min to pellet other organelles. The clear cytosolic fraction was collected in a fresh tube.

### Coimmunoprecipitation.

HSV-1-infected cells were incubated at a final concentration of 2% of formaldehyde for 1 h under gentle agitation at room temperature. The cross-linking step was stopped by addition of glycine at a final concentration of 0.2 M for 5 min at room temperature. Cells were centrifuged at 1,800 rpm for 5 min at 4°C and lysed in cell lysis buffer (Cell Signaling), supplemented with RNasin (40 U/mL; Promega) and PMSF (1 mM; Cell Signaling) on ice for 5 min. Cell lysates were sonicated three times on ice and centrifuged for 10 min. The clear cell lysate, already preclarified with protein A magnetic beads (Cell Signaling) according to the manufacturer’s instructions, was incubated with the antibody of interest overnight, i.e., IgG (conformation specific; L27A9), RIG-I (D33H10), or MDA5 (D74E4; Cell Signaling), at concentrations of 1/50 or 1/100, 1/50, and 1/100, respectively. Then, protein A magnetic beads were added to the lysate-antibody mix for 20 min at room temperature under agitation. After incubation, beads were harvested and washed three times. To avoid DNA contamination, beads were treated with Turbo DNase (Invitrogen) according to the manufacturer’s instructions and washed twice before elution. Elution was performed by addition of 0.2 M glycine at three times the volume of beads under agitation for 10 min. Using the magnetic separation rack, supernatant with proteins of interest was separated from beads and was neutralized by the addition of an equivalent volume of Tris-HCl (pH 8). This elution step was repeated twice.

### Western blotting.

Cells were treated with lysis buffer (0.5% Nonidet P-40, 20 mM Tris-HCl [pH 7.4], 120 mM NaCl, and 1 mM EDTA and EDTA-free protease inhibitors [Roche Applied Science]). Total protein samples were loaded on NuPAGE 4 to 12% bis-Tris gel (Invitrogen) and transferred to a nitrocellulose membrane (Invitrogen) according to the manufacturer’s instructions. Specific primary antibodies were incubated overnight, followed by HRP-linked secondary antibodies for 1 h according to standard protocols. Detection was performed with a chemiluminescence assay (Pierce). β-Actin HRP antibody (Sigma) diluted 1/50,000 was used to detect β-actin protein as a loading control. At 18 h postinfection, HSV-1-infected cells were tested for the proteins of the IFN pathway with antibodies from the RIG-I pathway antibody sampler kit (Cell Signaling) using dilutions recommended by the manufacturer. The expression of DNase-mCherry protein from plasmids was verified with anti-mCherry antibody (Abcam) used at 1/500. SPA tag detection of HEK-293T transfected cells or stable THP1 UL98-SPA- or UL12.5 SPA-expressing cell lines was performed using anti-Flag HRP-coupled antibody (Sigma) diluted 1/5,000. Depletion of the mtDNA in EtBr-treated and nontreated THP-1 cell lines was controlled using anti-TOMM20 (Abcam), as a marker of mitochondria, and anti-TFAM (Invitrogen) antibodies, as a marker of mitochondrial DNA, at 1/5,000 and 1/1,000, respectively. Purity of the whole-cell extract and the cytosol from the subcellular fractionation was controlled using anti-GAPDH (1/10,000; Sigma) as a marker of cytosol, anti-H2AX (1/1,000; Cell Signaling) as a marker of nuclei, and anti-TFAM (1/1,000) antibodies. Protein expression of UL12, UL12.5, and UL98 upon KOS37 SPA or KOS37 UL98-SPA infection at 12 h postinfection was verified with anti-Flag HRP-coupled antibody (1/5,000; Sigma).

### DNA extraction and quantitative PCR.

Total DNA from infected cells, from samples produced by the subcellular fractionation and from EtBr cell lines were extracted with the MasterPure purification kit (Epicentre Illumina Company). mtDNA was quantified using quantitative PCR based on TaqMan (Applied Biosystems) or based on SYBR green (Applied Biosystems). Conditions were 10 min at 95°C followed by 40 cycles of 15 s at 95°C, 15 s at 55°C, and 1 min at 68°C and were 2 min at 50°C and 10 min at 95°C followed by 40 cycles of 30 s at 95°C and 1 min at 60°C with a melting curve step, respectively. Data were normalized to the expression levels of the nuclear reference gene *β2M*. Primers are described in Table S1.

### Real-time PCR.

All RNA was extracted from infected cells using the RNeasy Plus minikit (Qiagen), and RNA from coimmunoprecipitated eluted samples was harvested using the standard protocol of RNA precipitation with 3 M sodium acetate, 100% ethanol, and 1 μL of GlycoBlue (Applied Biosystems). cDNA synthesis was performed using QuantiTect reverse transcription kit (Qiagen 205311) from 50 ng to 1 μg of RNA. Expression of *APOBEC3s*, *IFN-α*, and *IFN-β* genes was essayed by real-time PCR based on TaqMan (Applied Biosystems) along with *RPL13A* and *HPRT1* as reference genes. Primers for the amplification of *A3* as well as for the quantification of mtDNA were previously described (24, 59). The other primers and probes are presented in Table S1.

### Deep sequencing.

For the analysis of A3-induced mutations in mitochondrial DNA, cells were infected with HSV-1 for 18 h. Total DNA from cytosolic fractions was extracted, and the *MT-COI* gene was amplified by standard PCR using the primers dHCoxI fwd and dHCoxI rev (Table S1). For the *MT-COI* gene, conditions were 5 min at 95°C, then 35 cycles of 30 s at 95°C, 30 s at 60°C, and 1 min at 72°C, followed by 10 min at 72°C. PCR products were purified with the NucleoSpin gel and PCR clean-up kit (Macherey-Nagel) and quantified using the Quant-iT dsDNA assay kit (high sensitivity; Invitrogen) according to the manufacturer’s instructions. The preparation of the DNA library was performed with the NEBNext DNA library preparation kit and NEBNext multiplex oligonucleotides for Illumina (New England Biolabs) for the fragmentation and multiplex-adapter ligation steps. Deep sequencing was performed with Illumina cBot and GAIIX technology.

Sequenced reads were cleaned from adapter sequences and trimmed using Trim Galore! 0.4.3 with a quality Phred score cutoff of 28 and a minimum length of 20 (www.bioinformatics.babraham.ac.uk/projects/trim_galore), and quality was assessed using FastQC (www.bioinformatics.babraham.ac.uk/projects/fastqc). Sequencing reads were subjected to BLAST search using BLASTn (60, 61) with the following parameters: -task dc-megablast -perc_identity 20 -max_target_seqs 1 -outfmt “6 qseqid qstart qend sstart send qseq sseq evalue bitscore score length mismatch gaps qframe sframe.” Output was analyzed using a homemade python script (available on request). Further analyses were performed with R version 3.6.0 and R studio, as well as the Bioconductor packages ggplot2 and reshape2.

### Immunofluorescence.

THP-1 cells were plated and infected in 4-well chamber poly-l-lysine glass slides (Lab-Tek, Thermo Scientific). Cells were washed with phosphate-buffered saline (PBS), fixed in 4% paraformaldehyde, and permeabilized by incubation in 50/50 acetone-methanol (Sigma). Mouse monoclonal anti-ICP8 antibody (Life Technologies) and rabbit monoclonal anti-TOMM20 antibody (Abcam) were incubated at 1/750 and 1/250, respectively, in PBS-bovine serum albumin (BSA; 0.5%) for 1 h at room temperature. Secondary antibodies Alexa Fluor 488 goat anti-mouse and Alexa Fluor 647 goat anti-rabbit (Invitrogen) were both used at 1/750 for 45 min at room temperature, protected from the light. SPA tag detection in UL98-SPA- and UL12.5-SPA-expressing THP-1 cell lines was performed using Cy3-coupled anti-Flag antibody (Sigma) diluted at 1/100. Nuclei was stained with DAPI (1 μg/mL; BD Biosciences). Slides were mounted with the glycerol-based medium SlowFade Diamond antifade mountant (Invitrogen). Imaging was performed with a Leica SP8 confocal microscope. Both EtBr-treated and nontreated THP-1 cell lines were fixed and permeabilized in 4-well chamber poly-l-lysine glass slides as previously described (37–39). Rabbit monoclonal anti-TOMM20 antibody (Abcam) was used at 1/250 to stain mitochondria, and mouse monoclonal anti-TFAM antibody (Invitrogen) was used to stain mtDNA at 1/50 for 1 h. Secondary antibodies Alexa Fluor 488 goat anti-rabbit and Alexa Fluor 633 goat anti-mouse immunoglobulin (Invitrogen) were both used at 1/750 for 45 min at room temperature. Slides were mounted with Fluoromount-G medium, with DAPI (Invitrogen). Imaging was performed using a Leica SP5 confocal microscope. Transfected HeLa cells with pcDNA 3.1 DNase-mCherry were washed with PBS, fixed in 4% paraformaldehyde, and directly mounted with Fluoromount-G medium, with DAPI (Invitrogen). Imaging was performed using a Leica DM IRB microscope. All images were analyzed using Fiji software ImageJ version 1.52p.

### Mitochondrial behavior analysis.

All images were deconvolved with Huygens Professional version 19.04 (Scientific Volume Imaging, The Netherlands), using the CMLE algorithm, with stop criteria when quality was 0.05 or signal-to-noise ratio (SNR) was 20 or when a maximum of 40 iterations was reached. From the deconvoluted images, the nucleus and mitochondrion signals were processed separately to segment the different structures present. The nucleus channel was first smoothed using a 3- by 3- by 3-pixel median filter in order to remove the deconvolution artifacts and then thresholded using the Otsu algorithm (62). Finally, a morphological opening operation with a sphere element with a radius of 1 was used to remove possible small remaining salient structures from the binary image. The mitochondrion channel was thresholded using the Otsu algorithm. The different mitochondria were identified using a connected-component object labeling algorithm, which detects connected regions in the binary volume. For more precision, all structures under 10 pixels were removed for the analysis. Each region was then considered mitochondria, and several quantifications were computed from them.

The statistical analysis of the impact of HSV-1 infection on the mitochondrial network was performed using three characteristics: (i) the mitochondrial fraction count (MFC) (63), (ii) the total mitochondrial volume (MV) for the cell, normalized to the nucleus volume, and (iii) the mitochondrial shape through the computation of sphericity, which indicates that an object’s shape is close to a sphere when close to 1 and the opposite when close to 0. Both MFC and MV were computed per cell, allowing comparison using category plots. Sphericity was observed per mitochondrion directly. As we may be dealing with small objects that do not apply any noise filtering on the image, we were able to observe a large number of objects with a sphericity of ≥1. This phenomenon is due to the discretization of the values used to compute the sphericity, mainly the surface calculation when the particle size is drastically low. The particles were removed from the analysis and considered either noise or debris.

### Flow cytometry analysis.

For DNA double-strand break analysis, at 18 h after HSV-1 infection, cells were washed with PBS, fixed in 2 to 4% ice-cold paraformaldehyde (Electron Microscopy Sciences) for 10 min, and permeabilized in 90% ice-cold methanol (Sigma) for 30 min. After PBS washing, cells were incubated with 1:100 diluted Alexa Fluor 647 mouse anti-γH2A.X (pS139) antibody (BD Pharmingen) in PBS–5% BSA for 1 h. Mitochondrial quantification was performed by staining EtBr-treated and nontreated THP-1 cells with the rabbit monoclonal anti-TOMM20 antibody (Abcam) diluted 1/40 in PBS–5% BSA for 1 h. After PBS washings, cells were incubated with 1/100-diluted Alexa Fluor 488 goat anti-rabbit in PBS–5% BSA for 45 min. For apoptosis analysis, HeLa and THP-1 cells were collected and washed with PBS, stained with 7-AAD viability staining solution (1/40; eBioscience), then washed with annexin V binding buffer (eBioscience), and stained by anti-annexin V eFluor 450 (1/20; eBioscience). Treatment with 100 μM etoposide in dimethyl sulfoxide was used as positive control. All stained samples were acquired on a MACSQuant analyzer (Miltenyi Biotech), and data were analyzed with FlowJo software (Tree Star, Inc.; version 8.7.1).
