# Supplementary figures and images for: Herpes Simplex Virus Type 1 Infection Disturbs the Mitochondrial Network, Leading to Type I Interferon Production through the RNA Polymerase III/RIG-I Pathway
Source: mBio. 2021 Nov 23;12(6):e02557-21. doi: 10.1128/mBio.02557-21 (PMC8609356; doi:10.1128/mBio.02557-21)

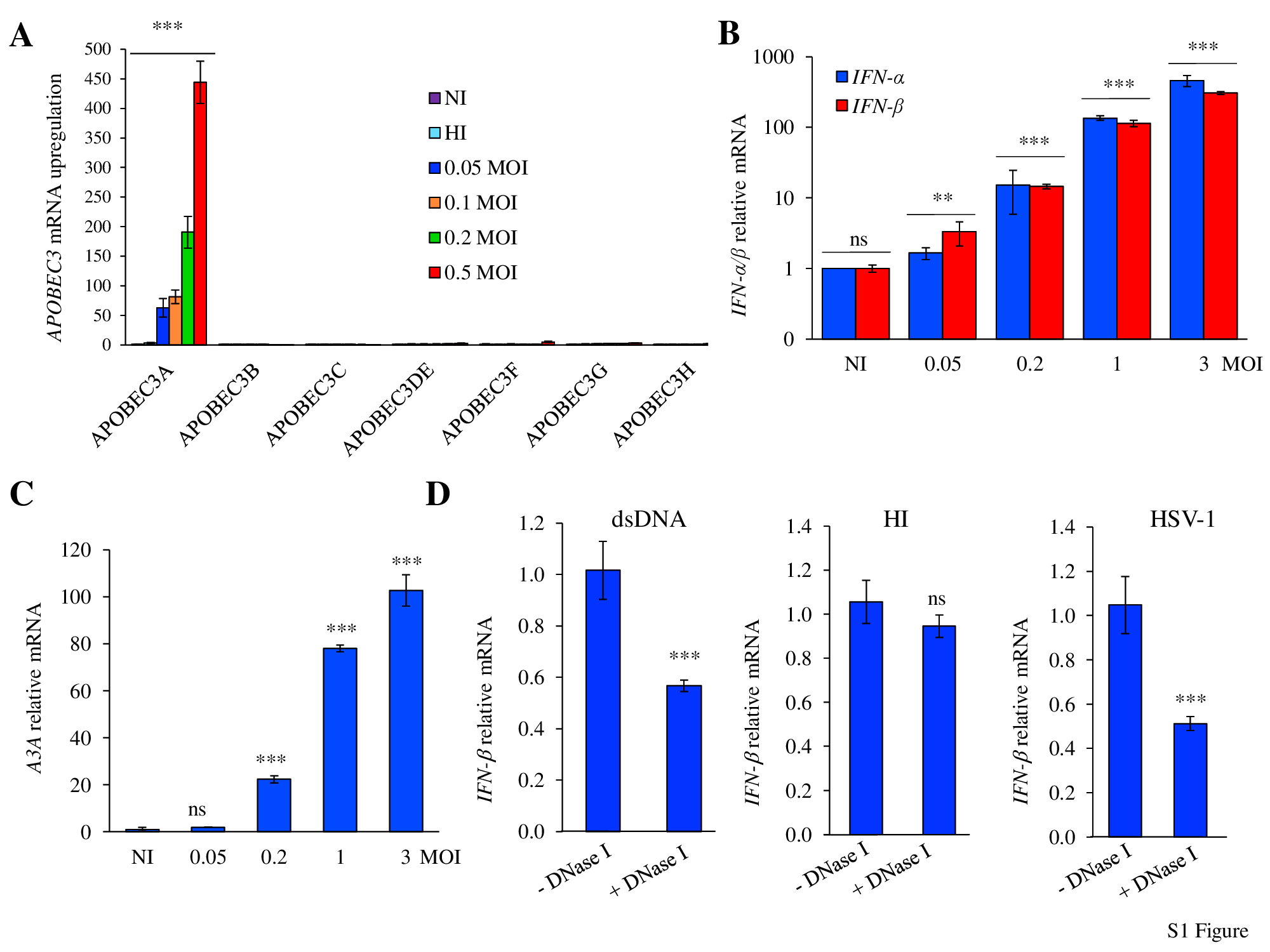

Supplement: FIG S1 [file mbio.02557-21-sf001.tif]

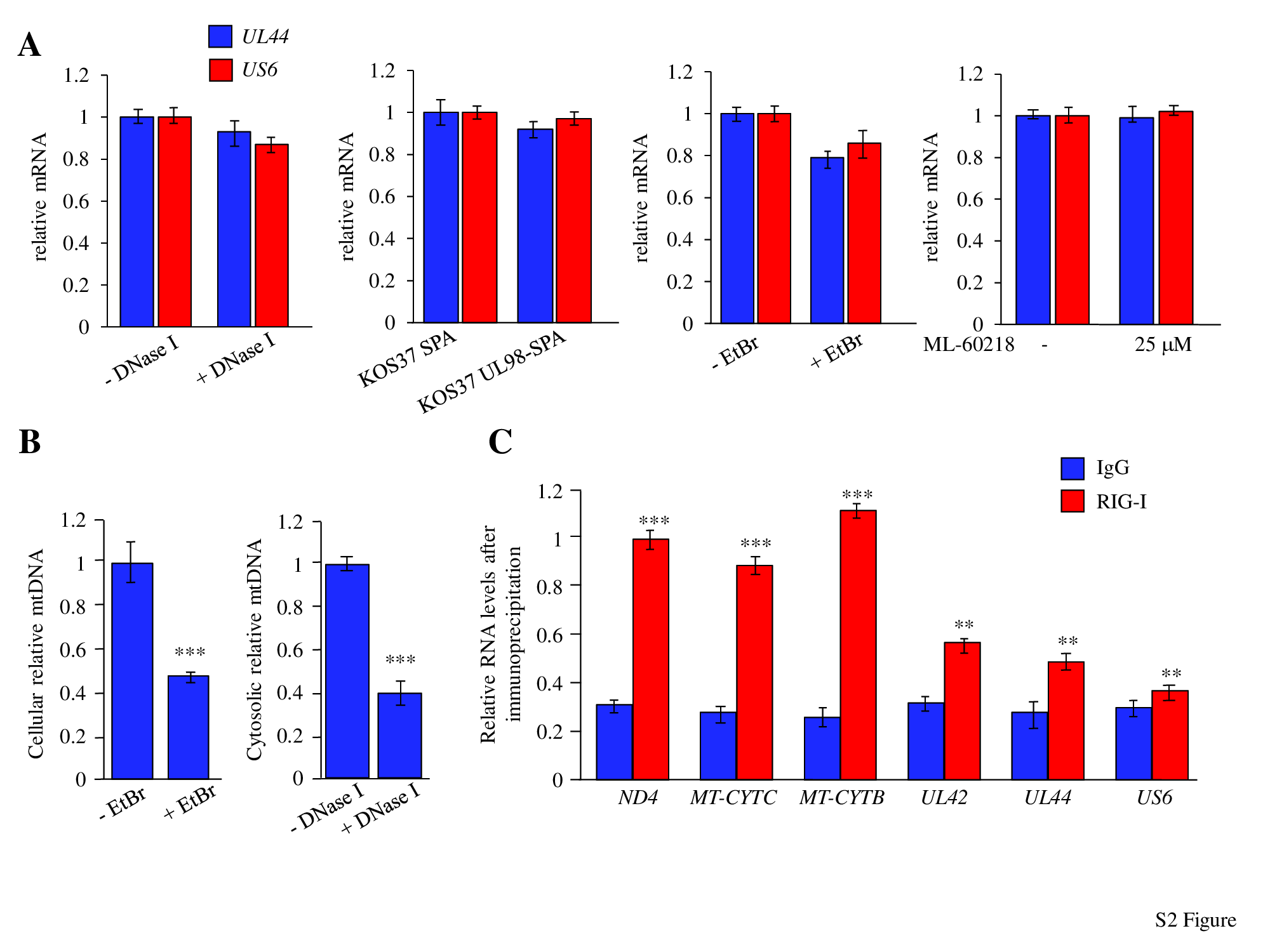

Supplement: FIG S2 [file mbio.02557-21-sf002.tif]

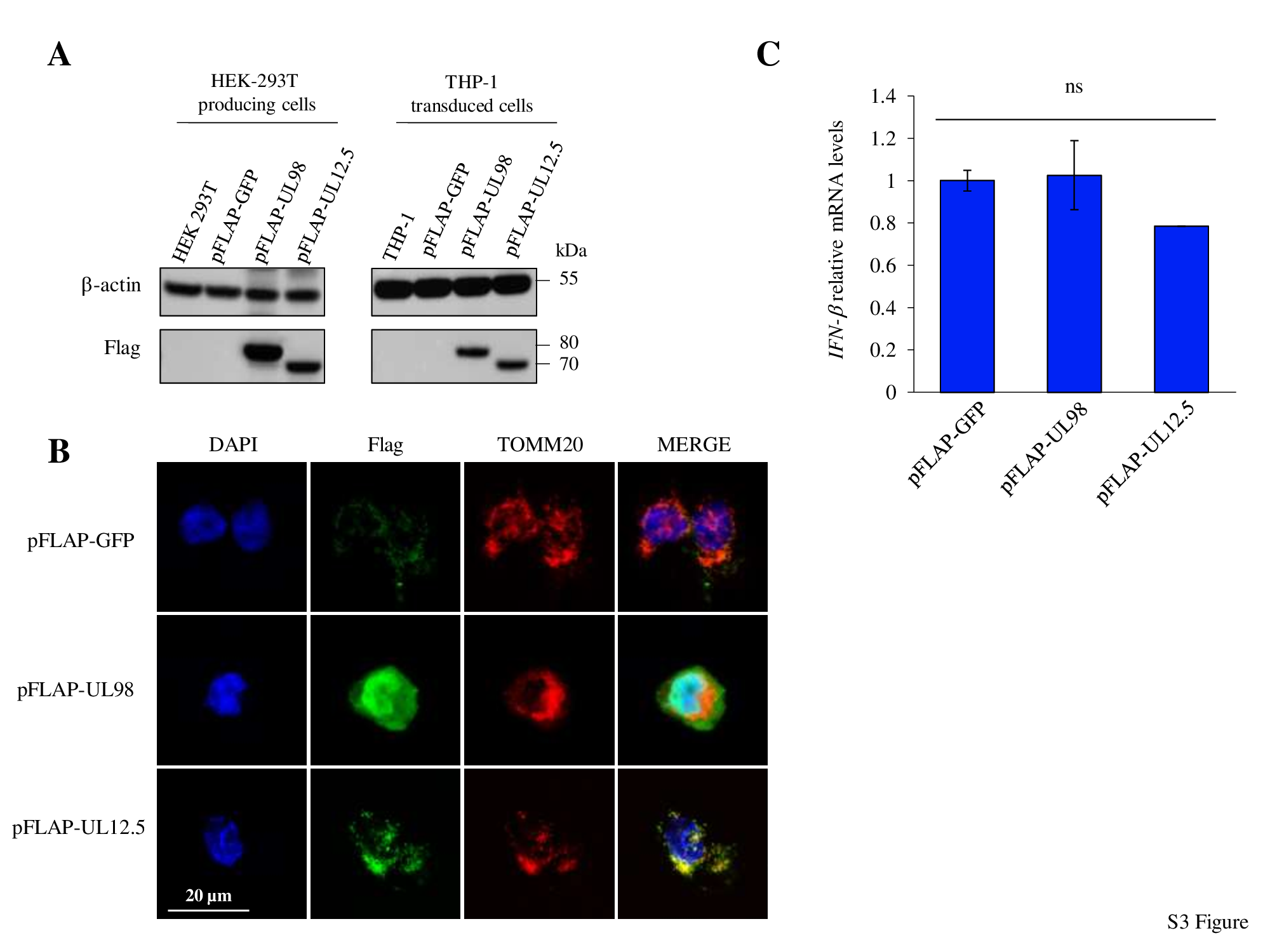

Supplement: FIG S3 [file mbio.02557-21-sf003.tif]

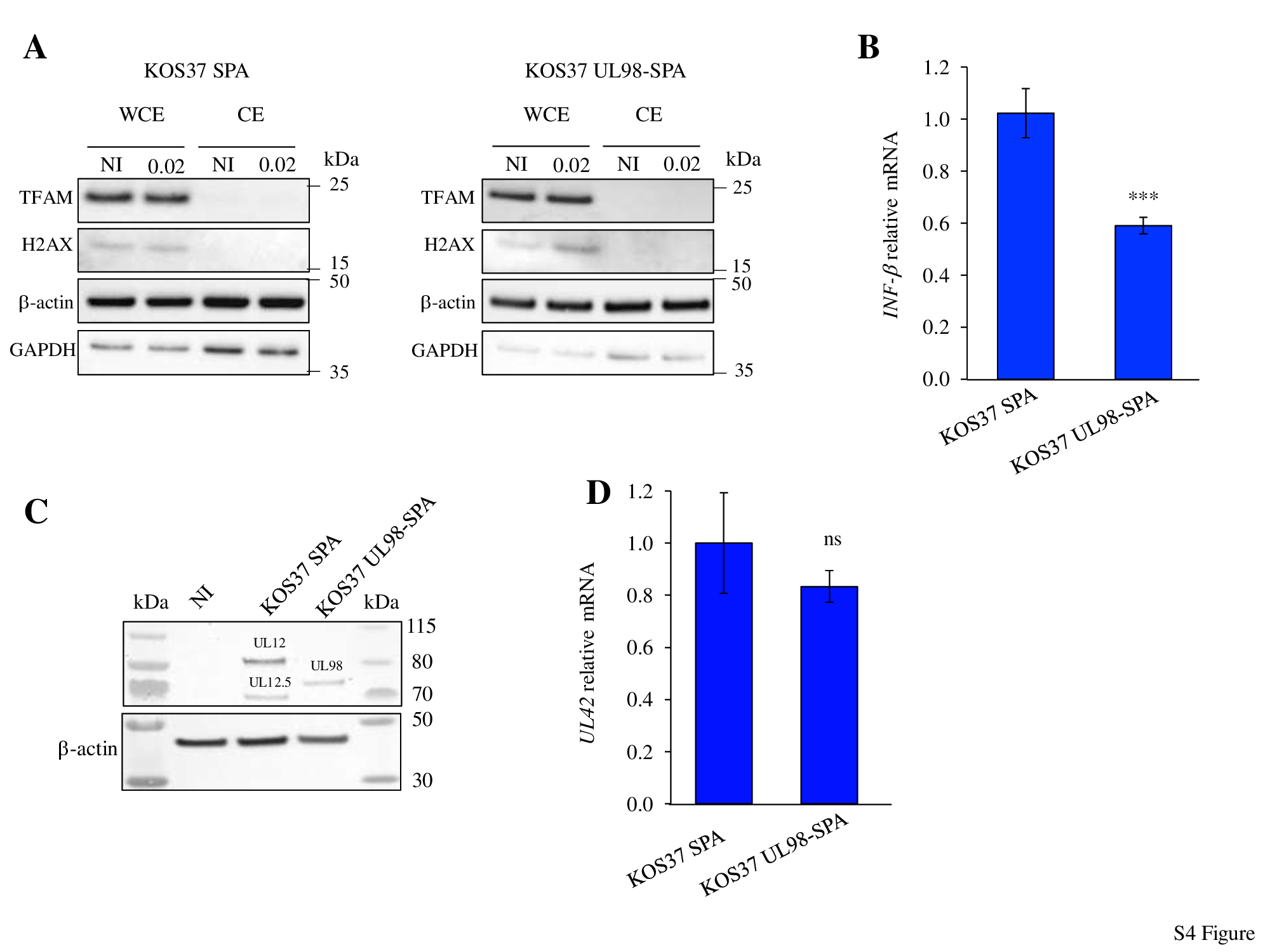

Supplement: FIG S4 [file mbio.02557-21-sf004.tif]

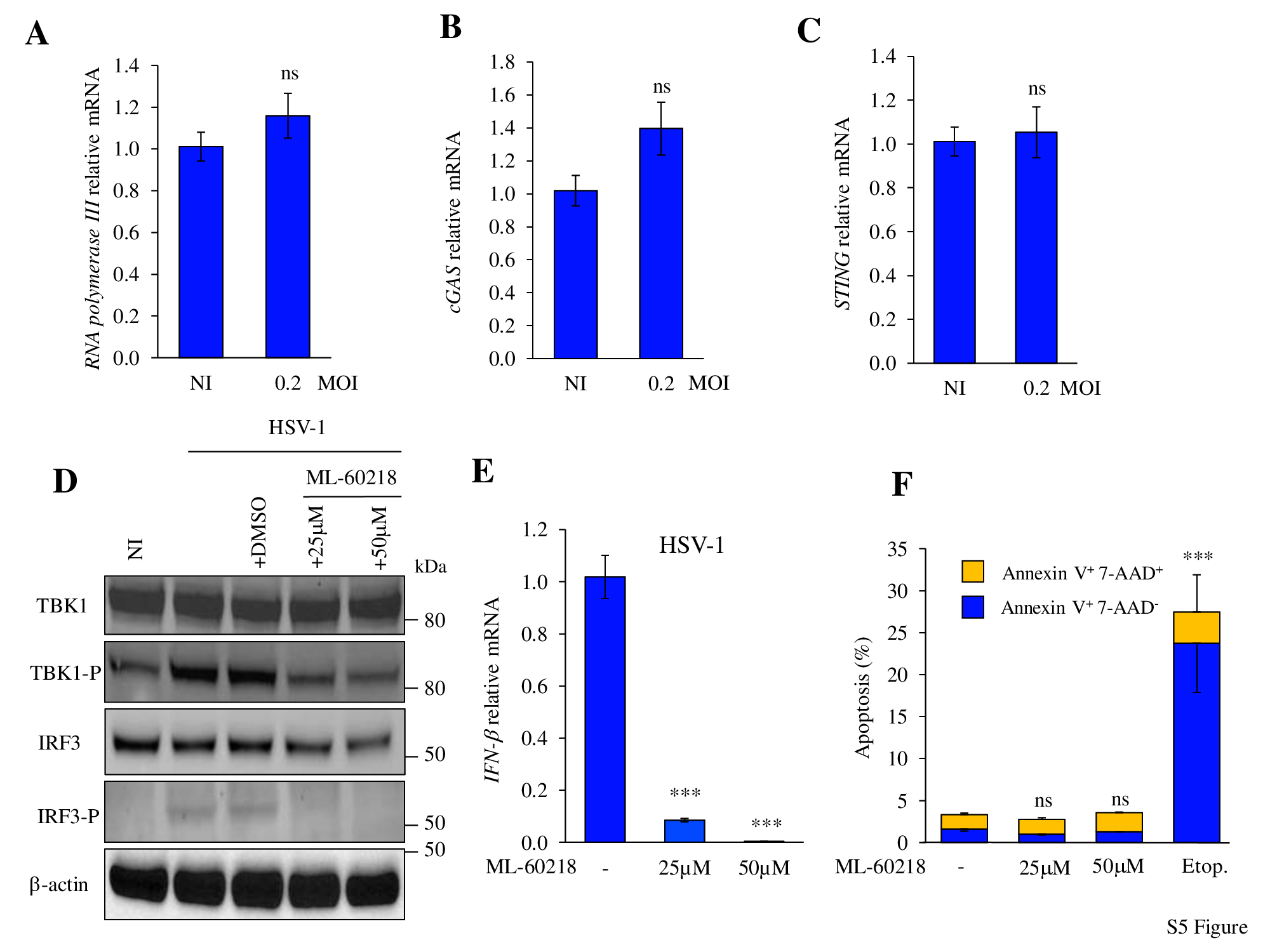

Supplement: FIG S5 [file mbio.02557-21-sf005.tif]

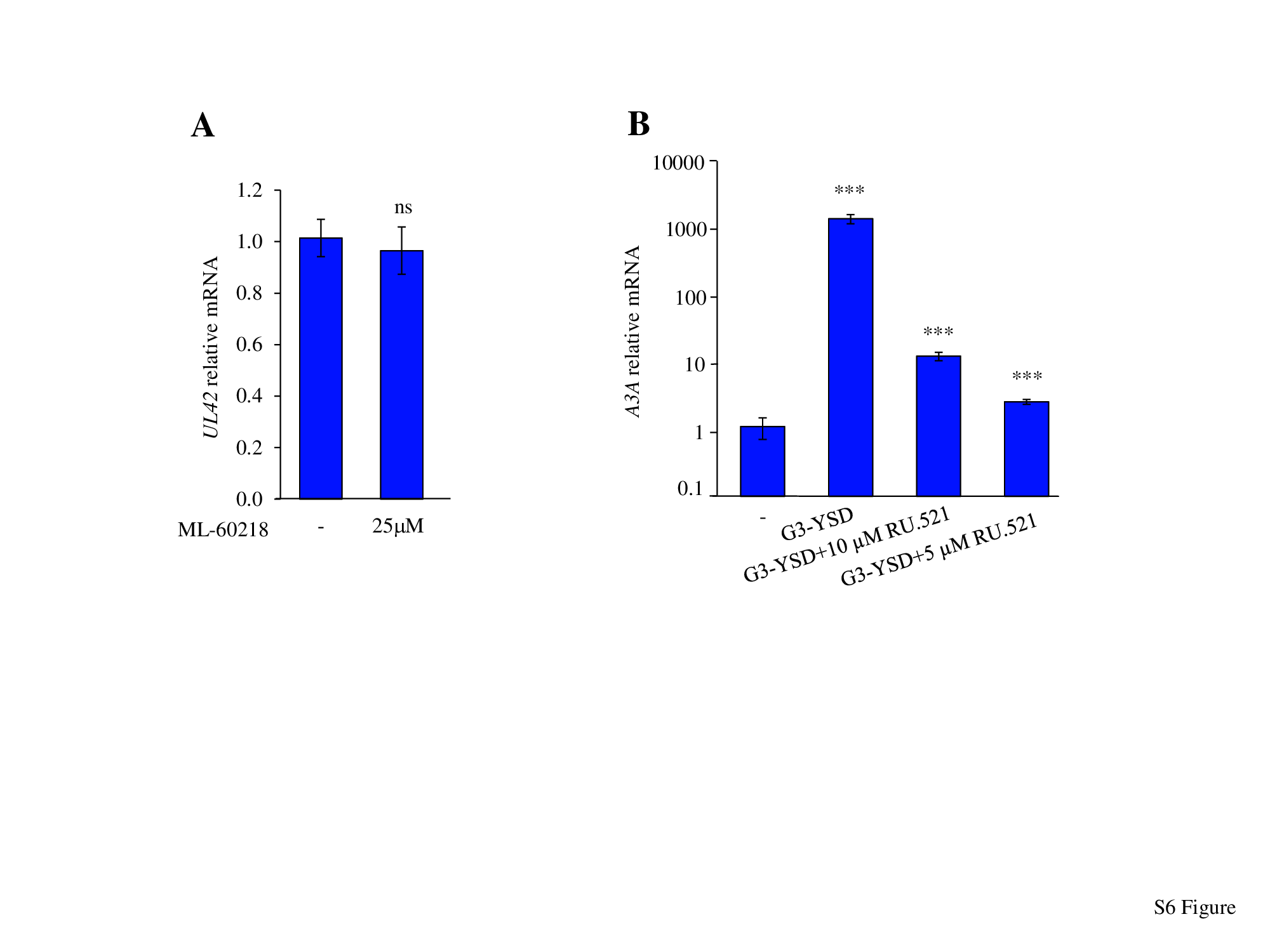

Supplement: FIG S6 [file mbio.02557-21-sf006.tif]
