# Supplementary material for: Herpes Simplex Virus Type 1 Infection Disturbs the Mitochondrial Network, Leading to Type I Interferon Production through the RNA Polymerase III/RIG-I Pathway
Source: mBio. 2021 Nov 23;12(6):e02557-21. doi: 10.1128/mBio.02557-21 (PMC8609356; doi:10.1128/mBio.02557-21)
Supplement: TABLE S1 [file mbio.02557-21-st001.docx]

**Primer Sequences Probes**

________________________________________________________________________________________________

*Rig-I (DDX58)* fwd 5’CTTTTTCTCAAGTTCCTGTTGGA *UPL79*

*Rig-I (DDX58)* rev 5’TCCCAACTTTCAATGGCTTC

*HPRT1* fwd 5’TGACCTTGATTTATTTTGCATACC *UPL73*

*HPRT1* rev 5’CGAGCAAGACGTTCAGTCCT

*IFN-α* fwd 5’TCCTGCTTGAAGGACAGACA *UPL63*

*IFN-α* rev 5’TTTCAGCCTTTTGGAACTGG

*IFN-β* fwd 5’CTTTGCTATTTTCAGACAAGATTCA *UPL20*

*IFN-β* rev 5’GCCAGGAGGTTCTCAACAAT

*STING* fwd 5’GATATCTGCGGCTGATCCTG *UPL1*

*STING* rev 5’CACCCCGTAGCAGGTTGTT

*MDA5* fwd 5’GGCACCATGGGAAGTGATT *UPL20*

*MDA5* rev 5’GATGATGATATTCTTCCCTTCCA

*cGAS* fwd 5’GCTCAGACTGAAGTGCGACTC *UPL66*

*cGAS* rev 5’AATTCTGGGGACTTCCAGTTTA

*RNA pol III* fwd 5’GCTGGACAAGAGCAACAGC *UPL21*

*RNA pol III* rev 5’TCATCTGTGATATGTTAATGAAGGAAC

*qRPL13* fwd 5’ctggaccgtctcaaggtgtt *UPL74*

*qRPL13* rev 5’gccccagataggcaaactt

*β2M* fwd 5’AATCAGATGGGTGTAGATCAAGG *UPL15*

*β2M* rev 5’GTTTCCACCCCTTCCATTTT

*CoxI* fwd 5’CCTCCCTTAGCAGGGAACTAC *UPL1*

*CoxI* rev 5’CACCTGCTAGGTGTAAGGAGAAG

*dHCoxI fwd* 5’GCGGTTGACTATTCTCTACAAACCACAAA

*dHCoxI rev* 5’GGGGGTTTTATATTGATAATTGTTGTGATGAAA

*mt3212* fwd 5’CACCCAAGAACAGGGTTTGT

*mt3319* rev 5’TGGCCATGGGTATGTTGTTAA

*ND4* fwd 5’CCTCGCTAACCTCGCCTTA

*ND4* rev 5’GGAGAACGTGGTTACTAGCACA

*UL42* fwd 5’GCCAGCGAGACGCTGAT

*UL42* rev 5’ACGCAGGTACTCGTGGTGA

*UL42* probe 5’6-FAM-CGCGAACTGACGAGCTTTGTGGT-TAMRA

*UL44* fwd 5’GTGACGTTTGCCTGGTTCCTGG

*UL44* rev 5’GCACGACTCCTGGGCCGTAACG

*US6* fwd 5’AGCTTCAGCGCCGTCAGCGA

*US6* rev 5’GAGTTCTGGCTGCGTGGCGT

*UL12.5-BamH1*fwd 5’AGGATCCACCATGTGGTCGGCGTCGGTGATCC

*UL98-BamH1*fwd 5’AAGGATCCACCATGTGGGGCGTCTCGAGTTTGGA

*SPA-SGRD1*rev 5’TCGTCGACGCTACTTGTCATCGTCATCCTTGTAGTC

_______________________________________________________________________________________________

**S1 Table**

Compendium of primers used for PCR, qPCR and RT-PCR; qPCR was either done by SYBR green or TaqMan with given UPL probes.

Fwd: forward, rev: reverse.
